# High Throughput Sequencing of T Cell Antigen Receptors Reveals a Conserved TCR Repertoire

**DOI:** 10.1097/MD.0000000000002839

**Published:** 2016-03-11

**Authors:** Xianliang Hou, Chong Lu, Sisi Chen, Qian Xie, Guangying Cui, Jianing Chen, Zhi Chen, Zhongwen Wu, Yulong Ding, Ping Ye, Yong Dai, Hongyan Diao

**Affiliations:** From the State Key Laboratory for Diagnosis and Treatment of Infectious Diseases, Collaborative Innovation Center for Diagnosis and Treatment of Infectious Diseases, The First Affiliated Hospital, College of Medicine, Zhejiang University, Hangzhou (XH, CL, QX, GC, JC, ZC, ZW, YD, PY, HD); Beijing Genomics Institute (SC); and Clinical Medical Research Center, The Second Clinical Medical College of Jinan University (Shenzhen People's Hospital) (YD), Shenzhen, Guangdong, China.

## Abstract

The T-cell receptor (TCR) repertoire is a mirror of the human immune system that reflects processes caused by infections, cancer, autoimmunity, and aging. Next-generation sequencing has become a powerful tool for deep TCR profiling. Herein, we used this technology to study the repertoire features of TCR beta chain in the blood of healthy individuals.

Peripheral blood samples were collected from 10 healthy donors. T cells were isolated with anti-human CD3 magnetic beads according to the manufacturer's protocol. We then combined multiplex-PCR, Illumina sequencing, and IMGT/High V-QUEST to analyze the characteristics and polymorphisms of the TCR.

Most of the individual T cell clones were present at very low frequencies, suggesting that they had not undergone clonal expansion. The usage frequencies of the TCR beta variable, beta joining, and beta diversity gene segments were similar among T cells from different individuals. Notably, the usage frequency of individual nucleotides and amino acids within complementarity-determining region (CDR3) intervals was remarkably consistent between individuals. Moreover, our data show that terminal deoxynucleotidyl transferase activity was biased toward the insertion of G (31.92%) and C (27.14%) over A (21.82%) and T (19.12%) nucleotides.

Some conserved features could be observed in the composition of CDR3, which may inform future studies of human TCR gene recombination.

## Abstract 2

Supplemental Digital Content is available in the text

## INTRODUCTION

Most T cell receptors (TCRs) consist of α and β chain, while others include γ and δ chain. These highly variable T lymphocyte membrane proteins are able to recognize antigenic peptides presented on heterologous cells in the context of the major histocompatibility complex (MHC). Specificity for the recognition of countless diverse peptide-MHC (pMHC) complexes is provided by 3 complementarity-determining regions (CDRs) of the TCR—CDR1, CDR2, and CDR3. Structural studies have established that CDR1 and CDR2 are encoded by germline sequences, which largely fix the TCR to the MHC platform. While CDR3 is a highly polymorphic principal recognition site, which largely engages the solvent-exposed chains of the MHC-bound peptide.^1,2^ The CDR3 regions are generated by somatic rearrangement between noncontiguous variable (V), and joining (J) gene segments for the α and γ loci, and between V, diversity (D), and J segments for the β and δ loci.^3^ The existence of multiple V, D, and J gene segments in germline DNA allows for a large amount of distinct CDR3 sequences to be encoded. Furthermore, receptor diversity can be augmented by trimming and addition of nontemplate nucleotides at the V(D)J junction sites (N-diversity mechanisms).^4^ The resulting hypervariable sequences of CDR3 make the recognition of millions of pMHC antigens that have never been encountered before with an extreme degree of precision and specificity. Notably, somatic rearrangement is not entirely random. Analyses of correlations between multiple repertoires from different individuals have revealed much greater similarity than would be expected at random.^3,5–8^ For example, an analysis of the naive CD8+ T cell population revealed that between any 2 donors the overlap is ∼7000-fold greater than for a random repertoire built with a uniform distribution.^9,10^ However, the underlying mechanism that accounts for this overlap remains elusive and its elucidation will require further study. Additionally, to understand the significance of various T cell clones that have been identified and to quantify such clones in both normal and disease states, a better understanding of the clonal frequency distribution of the T cell repertoire in healthy individuals is needed.

Currently, advances in sequencing technology have permitted the interrogation of complex sequencing targets at an unprecedented depth and a reasonable cost.^11,12^ Herein, we used high-throughput sequencing technology to measure the following features of the overall TCR repertoire of 10 healthy donors: length of the CDR3, VD indel (insertion and deletion), and DJ indel; the clonal frequency distribution; V, D, and J segment usage frequencies; the usage pattern of nucleotides and amino acids in the CDR3 region; and the nucleotide insertion bias. This study aimed to obtain new quantitative insights into the adaptive immune system in health to gain a better understanding of clonal diversity and TCR gene recombination in humans.

## MATERIALS AND METHODS

### Clinical Samples

Peripheral blood samples were collected from 10 healthy donors who tested negative for anti-hepatitis B surface antigen (anti-HBsAg) antibodies and anti-HIV antibodies and exhibited no clinical or laboratory signs of other infectious diseases or immunological disorders. Among these 10 healthy donors, 5 were males and 5 were females. The patient cohort had a mean age of 32.16 ± 13.56 years, ranging from 19 to 45 years. Peripheral blood mononuclear cells (PBMCs) were prepared from whole blood treated with 5 mL of fresh EDTA-K2 anticoagulated by a Ficoll-Hypaque centrifugation (Pharmacia Biotec, Roosendaal, The Netherlands).^13^ This study was conducted in accordance with the tenets of the Declaration of Helsinki and was approved by the Ethics Committee of the First Affiliated Hospital, College of Medicine, Zhejiang University (Ref. No. 2015-313).

### T Cell Isolation and DNA Extraction

Informed consent was obtained from blood donors. Peripheral blood T cells were isolated with anti-human CD3 magnetic beads according to the manufacturer's protocol (Miltenyi Biotec, Bergisch, Gladbach, Germany).^14^ T-cell purity was shown to be >90% (data not shown), as determined by flow cytometry using mouse anti-human antibodies CD3-phycoerythrin (PE) (BD Biosciences, San Jose, CA). DNA was prepared from 0.5 × 10^6^ to 2 × 10^6^ T cells from each sample, which was sufficient for analyzing the diversity of TCR β-chain. DNA was extracted from T cells using GenFIND DNA (Agencourt/Beckman Coulter, Brea, CA) extraction kits following the manufacturer's instructions.

### Multiplex-PCR Amplification of the TCR-β CDR3 Region

The TCR-β CDR3 region was determined by finding the second conserved cysteine encoded by the 3′ position of the Vβ gene segment and the conserved phenylalanine encoded by the 5′ position of the Jβ gene segment,^3,9^ according to the criteria of the International Immunogenetics collaboration.^15^ To generate a template library for the Illumina HiSeq2000 instrument, multiplex PCR was designed to amplify rearranged TCR-β CDR3 regions from genomic DNA. The assay utilized a suite of 32 forward (F) primers that were each specific for a functional TCR-Vβ segment and 13 reverse (R) primers for the Jβ segment (Table 1). Universal forward and reverse primer sequences were contained at their 5′-ends of the forward and reverse primers, respectively, which were compatible with the GA2 cluster station solid-phase PCR.^9^ The PCRs (50 μL) were configured with 1× QIAGEN Multiplex PCR master mix, 1.0 μM VF pool (22 nM for each unique TCR Vβ F primer), 1.0 μM JR pool (77 nM for each unique TCR Jβ R primer, Shenzhen, China), 16 ng/μL gDNA, and 10% Qsolution (QIAGEN). The amplification protocol was as follows: 15 min at 95°C, 30 cycles of 30 s at 94°C, 30 s at 59°C, and 1 min at 72°C, followed by a final extension cycle of 10 min at 72°C on a PCR Express thermal cycler (Hybaid, California).^9^ In order to sample millions of recombinational TCR CDR3 loci, 12 to 20 wells of PCR were executed for every library. After amplification and separation by agarose gel electrophoresis, products were purified using a QIAquick PCR Purification Kit. The final library was quantified in 2 ways: by determining the average molecule length using an Agilent 2100 Bioanalyzer (Agilent DNA 1000 Reagents, California) and by real-time quantitative PCR (qPCR; TaqMan Probe, ABI, California). Libraries were amplified using cBot to generate clusters on the flow cell, and an amplified flow cell was pair-end sequenced using a HiSeq2000 instrument (Illumina, California), generally using a read length of 100 bp.

**TABLE 1:**
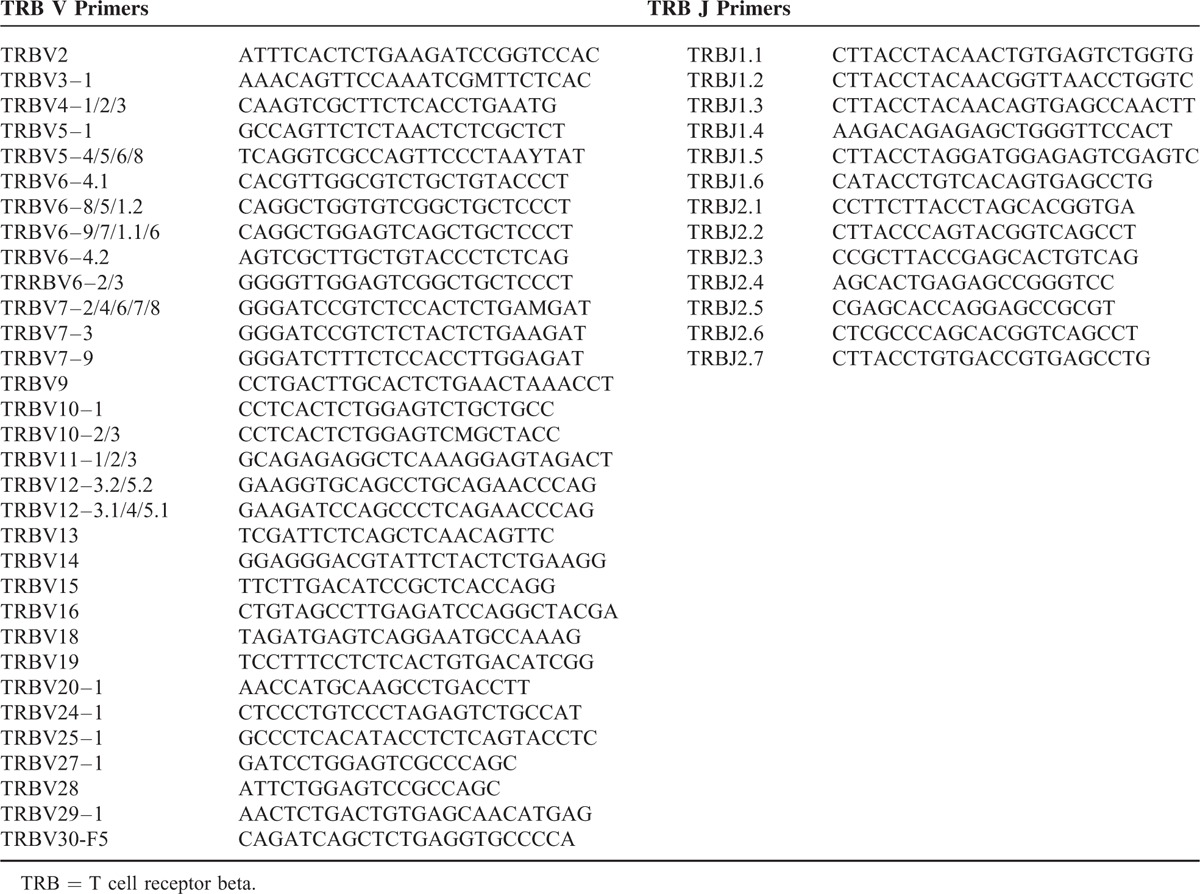
TRB V/J Primers

### High-Throughput Sequencing and Data Analysis

PCR products were sequenced using an Illumina Genome Analyzer, California. In the sequencing process, we used a well-defined human DNA fragment (internal control) in the control lane to monitor the sequencing quality. The DNA fragment is a small fragment (170–800 bp), which enables quick alignment and estimation of error rates. In addition, illumina cluster generation algorithms were optimized around a balanced representation of A, T, G, and C nucleotides. The quality of HiSeq sequencing ranged from scores of 0 to 40. This quality was included in the criteria for filtering out low-quality reads. The relationship between the sequencing error rate (E) and the sequencing quality (sQ) is shown in the formula: 
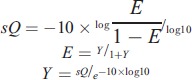


In addition, some common situations of sequencing error rate and sequencing quality correspondence are shown in Table 2.

**TABLE 2:**
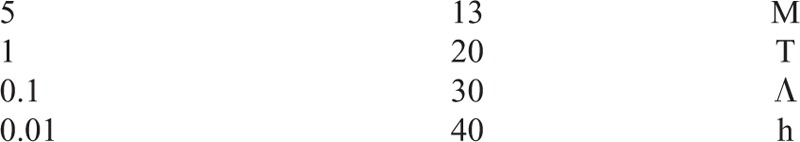
Correspondence Between Solexa Sequencing Quality and Error Rate

First, we filtered the raw data, including adapter contamination. Reads with an average quality score lower than 15 (based on the Illumina 0–41 quality system; the sequencing error rate is 3% when the quality score is 15) were removed, and a threshold for the proportion of N bases was set as <5% (sequences with higher values were removed). Next, a few bases with low quality (lower than 10) were trimmed; the quality score was expected to be >15 after trimming and the remaining sequence length was expected to be >60 nt. After filtering, pair-end (PE) read pairs were merged into a single-contig sequence by 2 steps: by aligning tail regions of 2 sequences and assessing the identity (using software developed by BGI, COPE v1.1.3) or sequences with at least 10 bp overlap and the overlapping section showing at least 90% base match; and as different primers might result in sequences of different lengths, some sequences might be very short (<100 bp), so all bases in these sequences would be analyzed and such reads would be merged by aligning the head part of the sequence (using software developed by BGI, FqMerger). In this manner, merged contig sequences and a length distribution plot were obtained.

For alignment, miTCR (developed by MiLaboratory; http://mitcr.milaboratory.com/downloads/) was used. This program has an automated adjustment mechanism for errors that are introduced by sequencing and PCR and can provide statistical data for alignments, such as CDR3 expression and indel. After alignment, the following method was used for sequence structural analysis: the number of each nucleotide and the proportion at each position was analyzed; according to the final position of the V gene, the start site of the D gene, the end site of the D gene, and the start site of the J gene after alignment were determined and the indel (insertion and deletion) that were introduced during V(D)J recombination were identified; and nucleotides were translated into amino acids. According to the identity of each sequence after alignment, the frequency of expression for each clone could be calculated. The expression of each distinct DNA sequence, amino acid sequence, and V–J combination was also identified. Additionally, to assess the diversity of each sample, the distinct clone number, Simpson coefficient, and Shannon–Waver coefficient were each calculated based on different resolutions of distinct DNA sequences, amino acid sequences, and V–J combinations. The frequency of expression of each sample was also calculated at different resolutions of distinct DNA sequences, amino acid sequences, and V–J combinations (Supplemental document 1). Moreover, a specific expression map was constructed and used to plot a heat-map according to the V–J combination profile (Supplemental document 2). TCR repertoire diversity was calculated based on the Simpson index of diversity (Ds)^16^ and the Shannon–Wiener index (H′).^17,18^

## RESULTS

Using high-throughput sequencing (Illumina Genome Analyzer), we sequenced the TCR-β repertoires from T cells collected from PBMCs of 10 healthy individuals, obtaining an average of 1.88 million total raw reads (pairs) per sample. After filtering, including the removal of adaptor sequences, contamination, and low-quality reads, we collected an average of 1.79 million reads, which met our quality requirements. Then, we used miTCR, which was developed by MiLaboratory, to map reads to available databases. We obtained an average of 87,125 unique CDR3 nucleotide sequences per sample after filtering out all identical redundant sequences within each sample. Additionally, we identified on average 83,448 unique CDR3 amino acid clonotypes and 1337 VJ combinations for each sample from 10 healthy individuals.^19^ Based on these data, it became apparent that a given TCR amino acid sequence could be encoded by many different nucleotide sequences (mean, 1.04).

### Distribution Characteristics of the Lengths of CDR3, VD Indels, and DJ Indels

**FIGURE 1:**
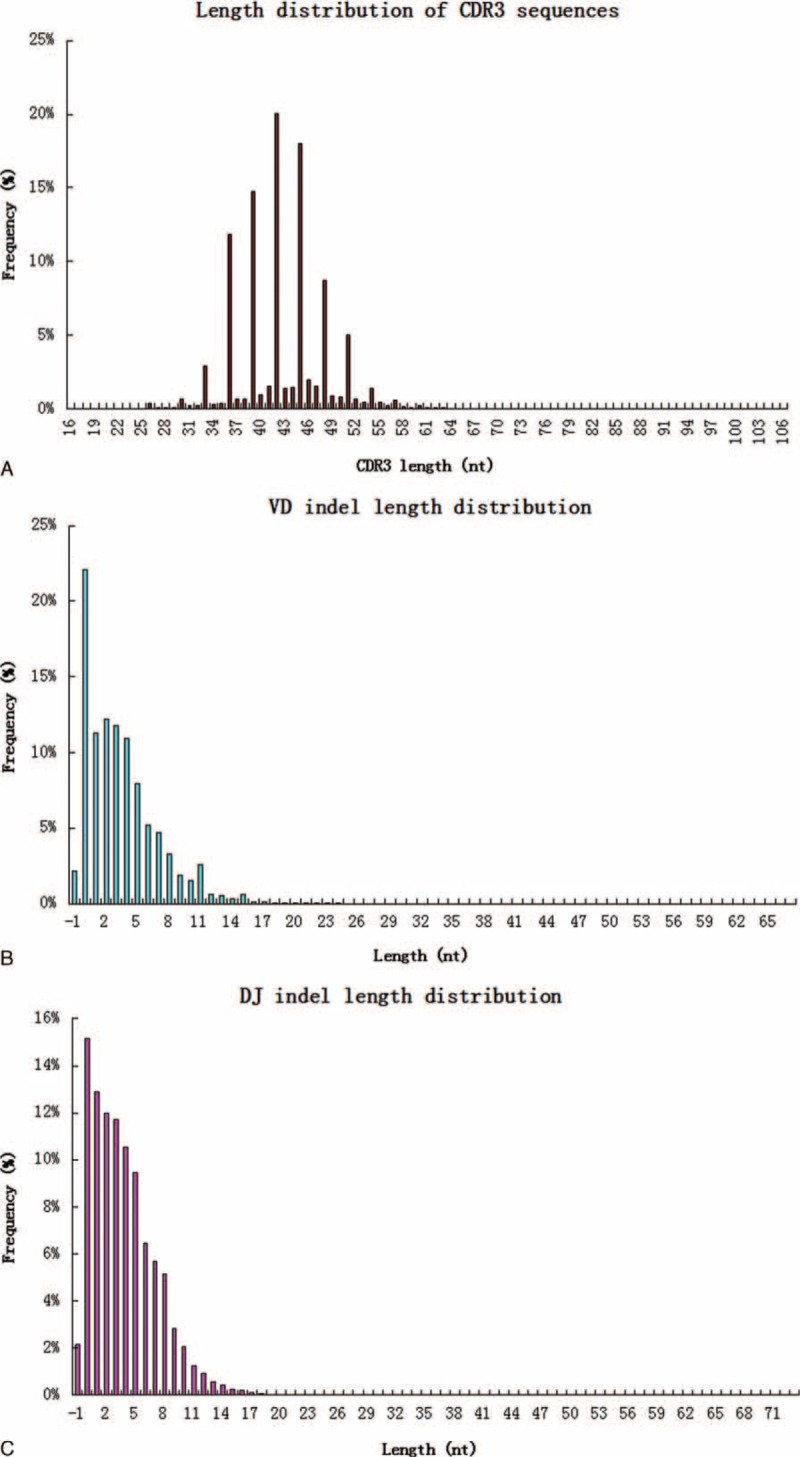
Length distribution of CDR3, VD indel, and DJ indel (average of 10 individuals). (A) Among 106,742,778 total high-quality sequences, the 5 most frequently observed CDR3 lengths were 42, 45, 39, 36, and 48 nt. (B) The 5 most frequent VD indel lengths were 0, 2, 3, 1, and 4 nt, respectively. (C) Regarding the number of (inserted + deleted) nucleotides at the Dβ–Jβ junctions, the 5 most common observed DJ indel lengths were 0, 1, 2, 3, and 4 nt, respectively. CDR = complementarity-determining region.

The length of the TCR CDR3 loop is an important determinant of T cell repertoire diversity. In this present study, we first assessed distributions in CDR3 length across the unique CDR3 sequences (regardless of clonal expansion) in human T cells from 10 healthy volunteers, and found that the average length of the CDR3 region was 43.9 bp, and the average length of total insertions for the Vβ–Dβ and Dβ–Jβ junctions was 8.4 bp. We then assessed the distributions in CDR3 length across the overall TCR-β repertoire (including the size of each clonotype). A total of 106,742,778 high-quality sequences were identified. The CDR3 length varied from 16 to 106 nt, with a peak at 42 nt, and the 5 most frequently observed CDR3 lengths, VD indel lengths (the number of [inserted + deleted] nucleotides at the Vβ–Dβ junction), and DJ indel lengths were identified (Figure 1).^19^

### Clonal Frequency Distribution of the T cell Repertoire

**FIGURE 2:**
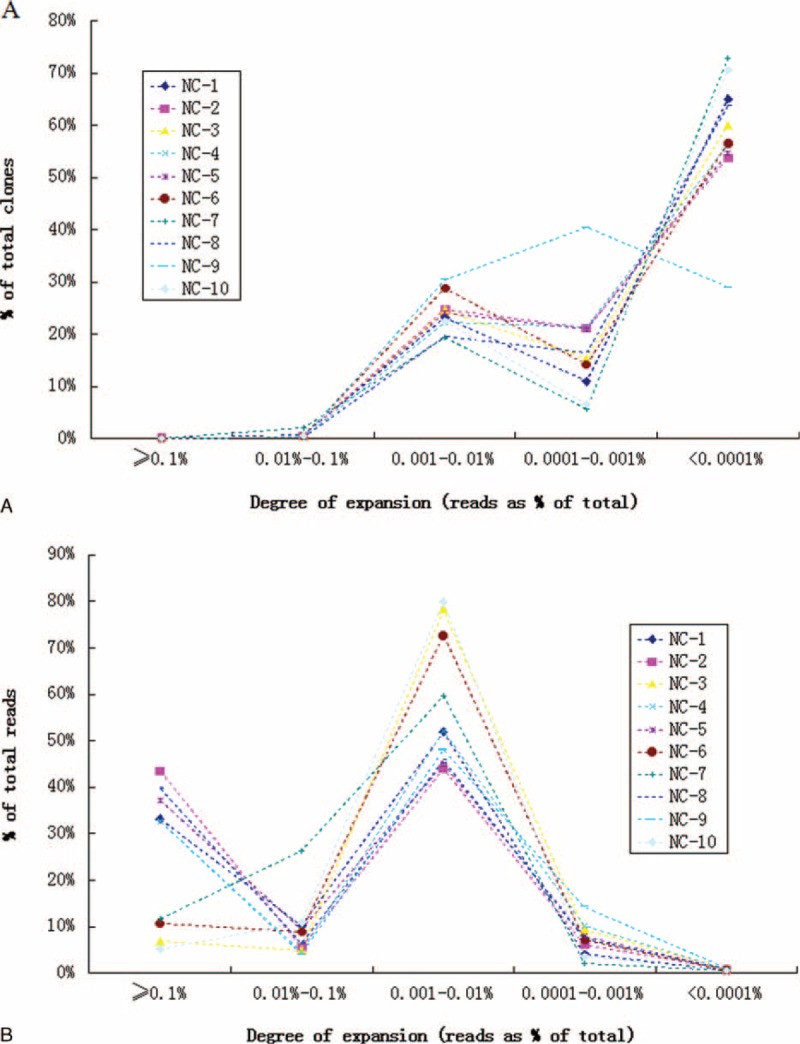
Degree of expansion and frequency distribution of T cell clones. (A) The frequency distribution shows a right-skewed distribution in which most clones were detected at a low frequency. (B) The frequency distribution shows that most of the repertoire was composed of a small number of HEC. The x-axis represents the degree of expansion. The y-axis represents the frequency (%) of each clone. HEC = highly expanded clone.

Based on the identity of each sequence after alignment, the frequency of expression of each clone could be calculated. Using this data, we estimated the degree of expansion of individual clones within the overall TCR repertoire based on the frequency of each unique CDR3 sequence within a sample (Figure 2). By statistical analyses, we found that clonotype abundance ranged from 1 to 2,836,517. Rare clonotypes, which were detected as single copies, represented on average 48.74% of all unique CDR3 sequence clonotypes per sample. Clones with a frequency of >0.1% of the analyzed TCR sequences were defined as highly expanded clones (HEC). Only 4.18 × 10^−2^% (range, 1.16 × 10^−2^–7.32 × 10^−2^%) of clones had expanded beyond this threshold. In absolute numbers, this frequency corresponded to a median of 33.3 clones (range 14–53). A summary of these HECs is presented in Supplemental Table 1. Subsequently, we determined the contribution of HECs to the overall T cell TCR repertoire. Strikingly, most of the repertoire could be accounted for by a small number of HECs. Using sample NC-2 as an example, a total of 43 HECs (6.59 × 10^−2^% of all the unique CDR3 sequence) accounted for 43.48% of the T cell sequences (total reads), whereas 53.73% (n = 35,051) of clones were very low-frequency clones (present at <0.0001% of all TCR sequences analyzed), which accounted for only 0.72% of the T cell sequences that were present (total reads).

### Sequence Composition of the CDR3 Regions

**FIGURE 3:**
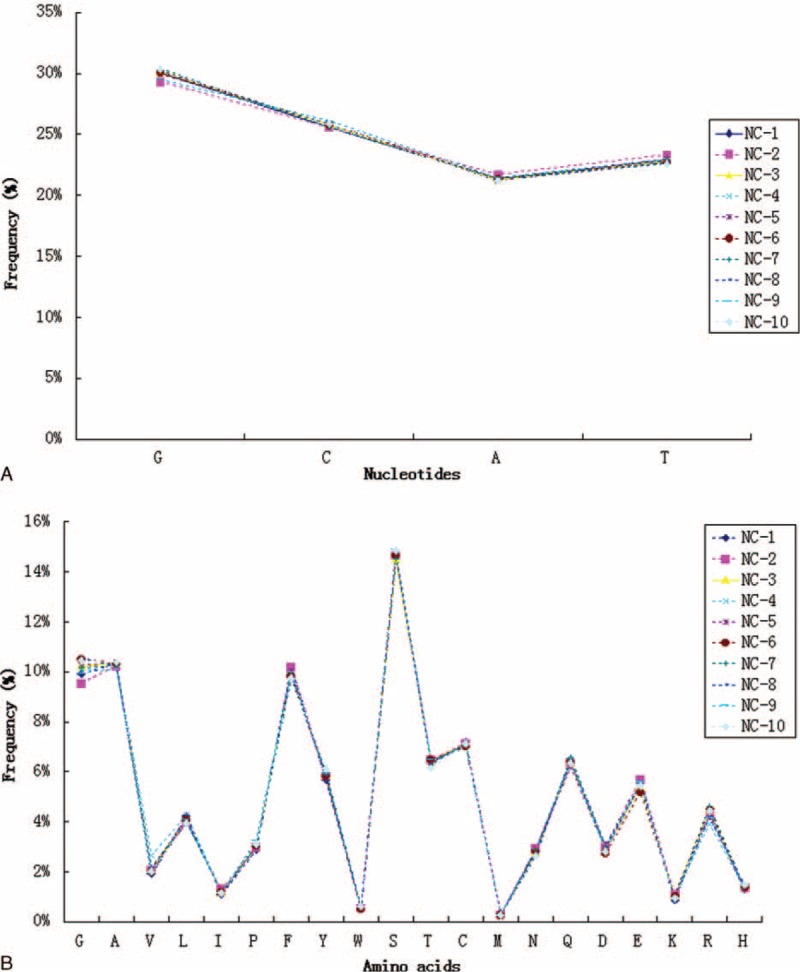
Nucleotide (A) and amino acid (B) composition of unique TCR-β clonotypes (irrespective of clonal expansion) identified in 10 healthy individuals. TCR = T cell receptors.

**FIGURE 4:**
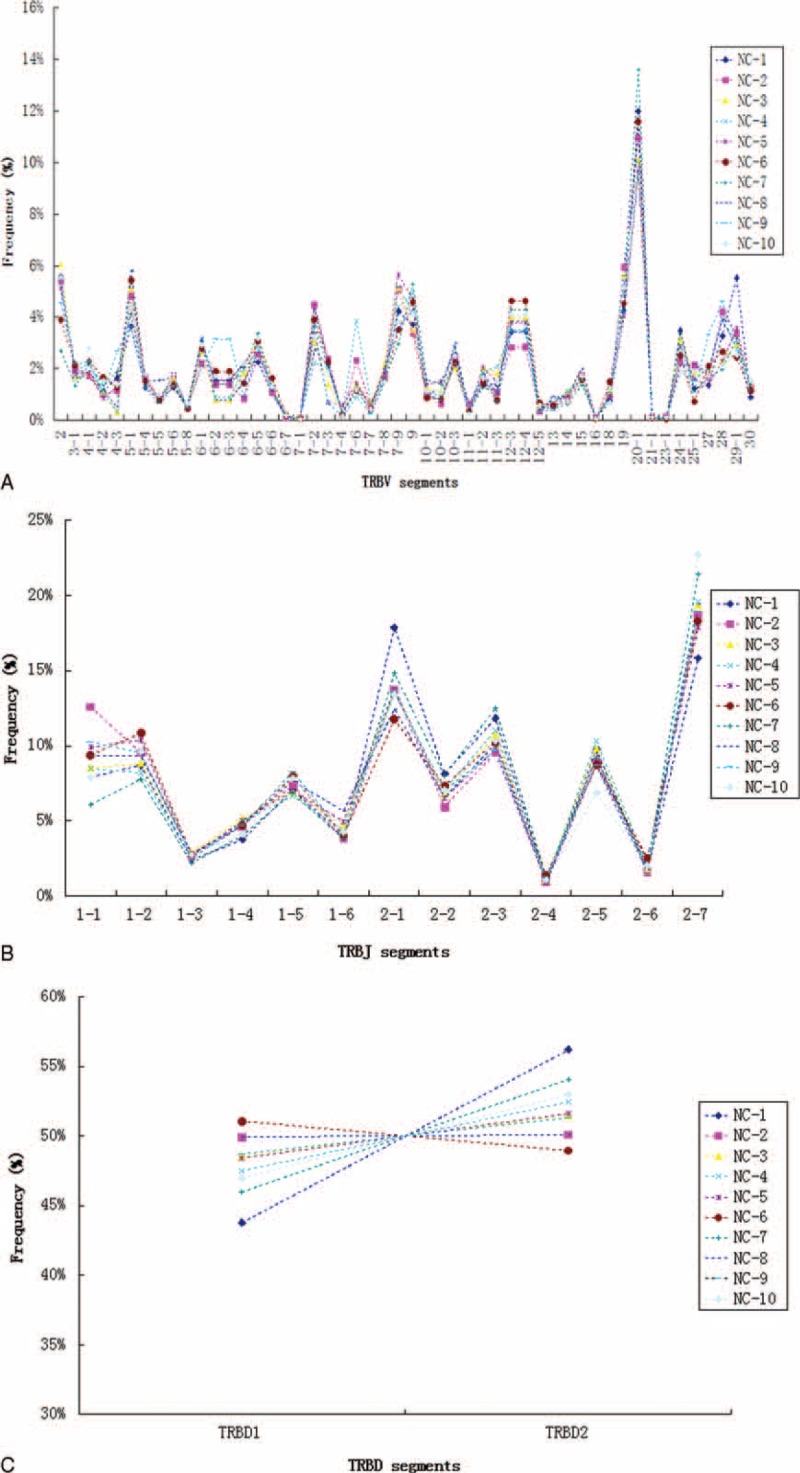
TRBV (A), TRBJ (B), and TRBD (C) repertoire analyses of unique TCR-β clonotypes found in PBMCs from 10 healthy individuals. PBMC = peripheral blood mononuclear cell, TCR = T cell receptors, TRBD = T cell receptor beta diversity, TRBJ = T cell receptor beta joining, TRBV = T cell receptor beta variable.

To avoid distortion of the dataset by dominant clones that had expanded as a consequence of an immune response, each unique CDR3 sequence was counted as “1,” irrespective of how many copies were detected when we examined patterns of nucleotide composition, amino acid usage in CDR3 intervals, or T cell receptor beta (TRB) (V/J/D) gene segment usage frequencies. As shown in Figure 3, the frequency of usage of individual nucleotides (Figure 3A) and amino acids (Figure 3B) within CDR3 intervals was remarkably consistent between individuals. Nucleotide usage ranged from 30% (G) to 21% (A) among the 4 bases (Figure 3A). At the protein level (Figure 3B), the most frequently used amino acid was Serine (Ser, S), which accounted for 14.7% of all amino acids, whereas Methionine (Met, M) accounted for only 0.28% of amino acids. Additionally, no significant difference in overall T cell receptor beta variable (TRBV), T cell receptor beta joining (TRBJ), or T cell receptor beta diversity (TRBD) gene usage was observed (Figure 4). The average usage of this set of 50 TBRV genes for these 10 individuals ranged from 10.9% for TRBV20–1 to 0.05% for TRBV7–1 (Figure 4A). For the 13 Jβ gene segments, usage ranged from 19.2% for TRBJ2–7 to 1.09% for TRBJ2–4 (Figure 4B). Finally, TRBD gene segment usage was found to be 48.1% for TRBD1 and 51.9% for TRBD2 (Figure 4C).

**FIGURE 5:**
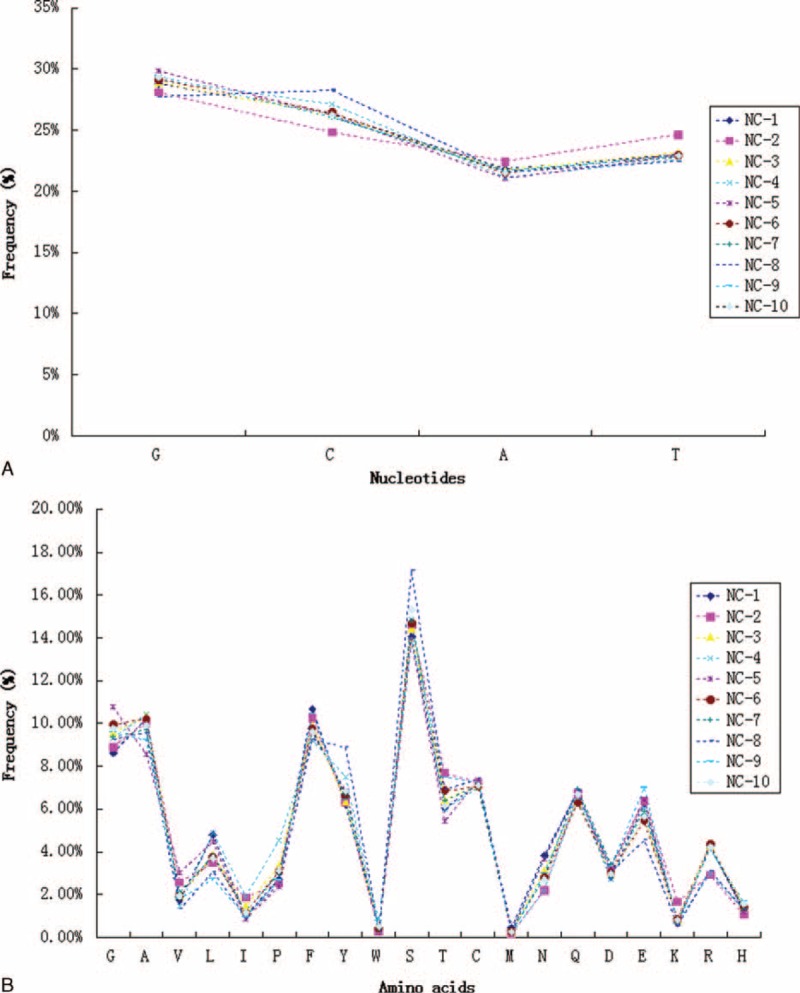
Nucleotide (A) and amino acid (B) composition of the total TCR-β repertoire in 10 healthy individuals. TCR = T cell receptors.

**FIGURE 6:**
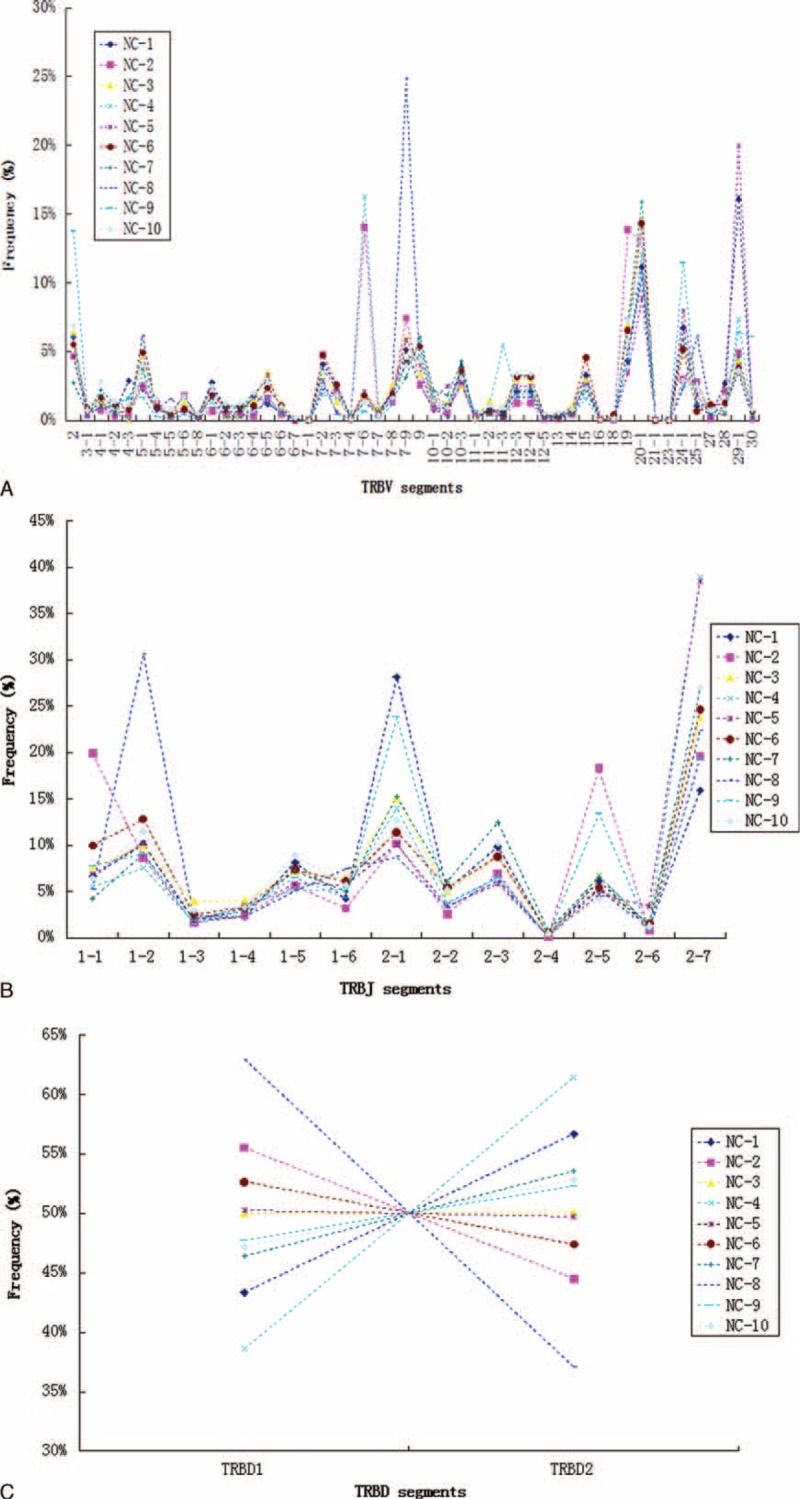
TRBV (A), TRBJ (B), and TRBD (C) repertoire analyses across the overall TCR-β repertoires of PBMCs from 10 healthy individuals. PBMC = peripheral blood mononuclear cell, TCR = T cell receptors, TRBD = T cell receptor beta diversity, TRBJ = T cell receptor beta joining, TRBV = T cell receptor beta variable.

However, our findings provide limited indications of the dominant repertoire features within the overall repertoires. Therefore, we also assessed patterns of nucleotide and amino acid usage in CDR3 intervals (Figure 5), as well as TRBV, TRBJ, and TRBD gene segment usage frequencies (Figure 6) across the overall TCR-β repertoires (including the size of each clonotype). Compared to the repertoire features of unique TCR-β clonotypes (irrespective of clonal expansion) that were observed in the 10 healthy individuals, repertoires among individuals were more variable for the total TCR-β repertoires. At the nucleotide level, the usage frequency of C bases between individuals ranged from 24.8% to 28.3% (Figure 5A). At the amino acid level, the usage frequency of S residues between individuals ranged from 13.9% to 17.2% (Figure 5B). However, the general trend remained similar among individuals for the usage pattern of nucleotides and amino acids. Regarding the degree of TCR-B (V/J/D) usage, the relationship of the frequencies of some TCRBV repertoires, such as VB7–9, VB29–1, VB7–6, VB2, and VB19, varied (Figure 6A), and the amount of variation was >10%. Notably, VB7–9 expression varied substantially among individuals (3.1–24.8%). Expression levels of the other Vβ segments appeared to be consistent among individuals (Figure 6A). Similarly, expression levels of several TCR-BJ segments were also variable among healthy individuals (Figure 6B), such as for BJ2–7 (16.0–39.0%), BJ1–2 (7.6–30.6%), and BJ (8.7–28.1%) (Figure 6B). Additionally, the frequency distribution diagram showed that TCR-BD segment usage was also altered (Figure 6C). The preponderance of these nucleotides, amino acids, and V(D)J gene segment usage frequencies among individuals were associated with the most dominant TCR-β clonotypes for each donor. For example, dominance of the TRBV7–9 and TRBJ1–2 clonotype (clonotype abundance up to 2,836,517, representing 23.44% of all assemblies) in the repertoire of donor NC-8 was largely responsible for the peak at the VB7–9 segment.

### Nucleotide Insertion Bias

**FIGURE 7:**
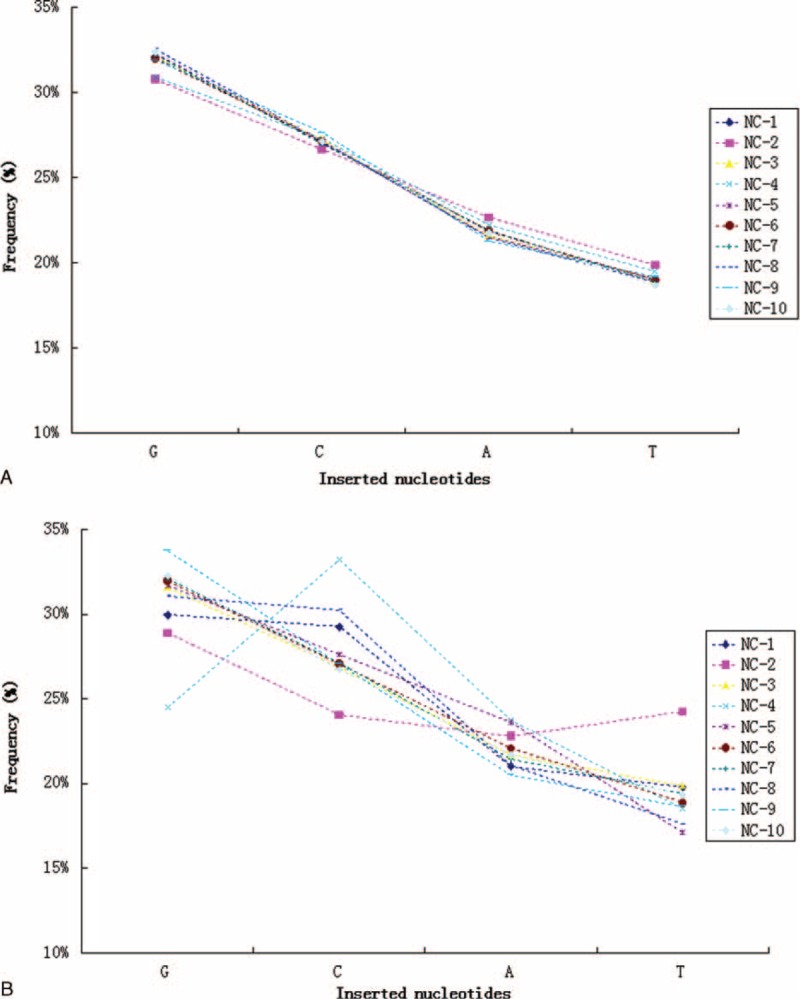
Tdt mononucleotide insertion bias. Mononucleotide frequencies of inserted junctional nucleotides at the V–D and D–J junctions of unique CDR3 nucleotide clonotypes (A) and the overall TCR-β repertoires (B) observed in 10 healthy individuals. CDR = complementarity-determining region, TCR = T cell receptors, Tdt = terminal deoxynucleotidyl transferase.

**FIGURE 8:**
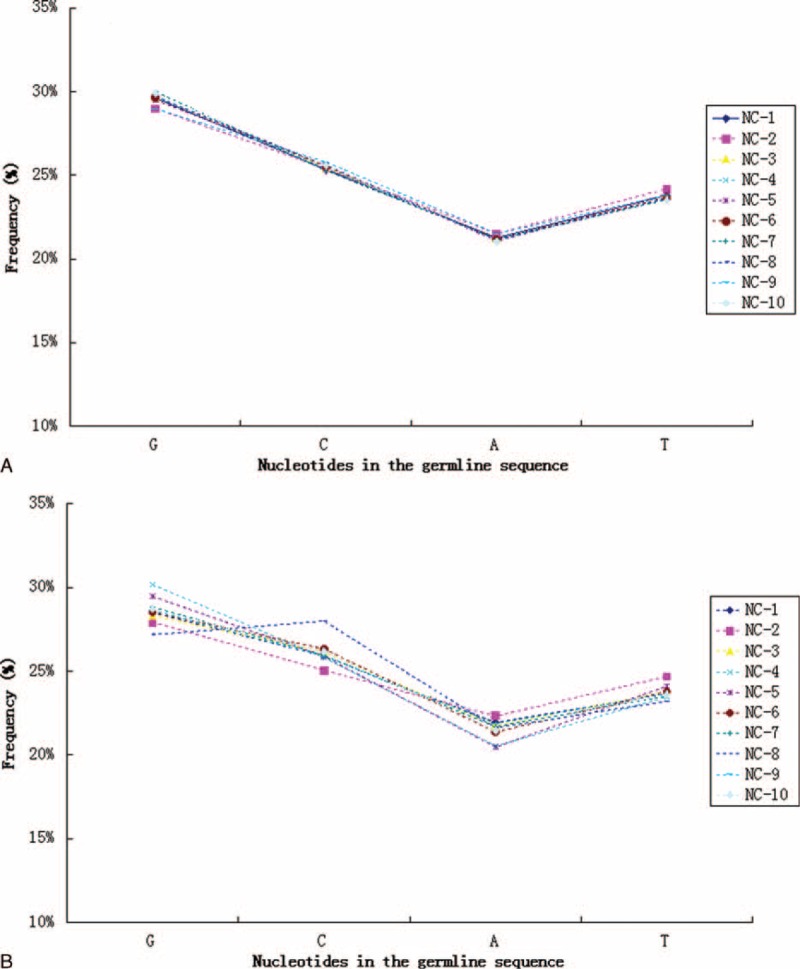
Mononucleotide frequencies in germline sequences of unique TCR-β clonotypes (A) and overall TCR-β repertoires (B) observed in 10 healthy individuals. TCR = T cell receptors.

Much of the CDR3 diversity in the TCR-β chains is created by the template-independent insertion of nucleotides at the Vβ–Dβ and Dβ–Jβ junctions by terminal deoxynucleotidyl transferase (Tdt). The frequency at which Tdt inserts each of the 4 nucleotides was been estimated (Figure 7). Similarly, for unique TCR-β clonotypes identified in each sample from 10 healthy individuals, the relative frequency of individually inserted nucleotides was found to be similar between individuals. Tdt was biased toward the insertion of G (31.92%) and C (27.14%) over A (21.82%) and T (19.12%) bases. Subsequently, we also assessed the nucleotide insertion bias across the overall TCR-β nucleotide repertoire (including the size of each clonotype), and found that the nucleotide insertion frequency was generally variable among individuals. Increased variance was associated with the most dominant TCR-β clonotypes for each donor. For example, dominance of the TGTGCCAGCAGCTTAG CGGGGGGGACCCCACCTATCTACGAGCAGTACTTC sequence (clonotype abundance up to 1,556,642, representing 15.04% of all assemblies) in the repertoire of donor NC-4 was largely responsible for the C nucleotide peak, as 6 C nucleotides were inserted in this sequence. Additionally, to further characterize the organization and recombination of the human TCR gene, we analyzed the nucleotide composition of the original germline sequences. As shown in Figure 8, the usage frequency of individual nucleotides within CDR3 intervals was indistinguishable between individuals. Similarly, compared to the mononucleotide frequency in the germline sequences of unique TCR-β clonotypes, repertoires among individuals were more variable for the overall TCR-β repertoires.

## DISCUSSION

T cells play central roles in host protection against infectious pathogens and can contribute to the development of autoimmune and allergic diseases. The ability of T cells to mount an immune response against diverse arrays of pathogens is primarily dependent upon the amino acid sequence of hypervariable CDR3 of the TCR, which is the special molecular structure that differentiates different populations of T cells. Herein, we used next-generation sequencing to investigate the characteristics and polymorphisms of the TCR CDR3 gene using T cells from 10 healthy volunteers. Most individual T cell clones were present at very low frequencies, which suggested that these clones had not undergone clonal expansion. Several HECs were present in each individual, which could have resulted from physiological responses to environmental antigens or pathogens. It was interesting to note that high-frequency TCRβ CDR3 sequences with fewer insertions and deletions have receptor sequences that are closer to the germline sequence.^7,9^ Additionally, we assessed the distribution of CDR3, VD indel, and DJ indel lengths among the 106,742,778 high-quality sequences, and the most common observed length was identified. It is known that different rearrangements can result in variable CDR3 lengths, and the characteristics of TCR clonality among different subfamilies can be determined by measuring lengths CDR3 subfamilies. Venturi et al^7^ found substantial differences in CDR3 length distributions between the memory and naive T cell pools. Moreover, accumulating evidence suggests that long IgH CDR3 loops are associated with self-reactive or polyreactive Abs,^20–23^ which may account for the removal of these cells from the repertoire during B cell development. Additionally, some studies have reported the expression of a truncated form of CDR3 by CD4 single-positive thymocytes,^24^ comparisons of the functions and characteristics of the long and short forms of CDR3 merit further study.

In this present study, we also systematically analyzed patterns of nucleotide and amino acid usage in CDR3 intervals, and the frequencies of V, D, and J segment usage in T cells from 10 healthy volunteers. Our data showed a general trend for the usage frequencies of the TRBV, TRBJ, and TRBD repertoires to be similar among individuals, which was in accord with previous studies. Robins et al^3^ found that the J gene segment usage pattern in 4 different T cell populations (CD4^+^CD45RO^−^CD45RA^+^CD62L^+^, CD4^+^CD45RO^+^, CD8^+^CD45RO^−^CD45RA^+^CD62L^+^, and CD8^+^CD45RO^+^) was relatively constant within a given donor. Moreover, work carried out by Kitaura et al^25^ reported that the TRAV and TRBV repertoires in the common marmoset were conserved at the levels of individuals, tissues, and T cell subpopulations. Abnormal TRB (V/D/J) family usage might be associated with immune-mediated diseases. Sun et al^26^ detected a significant association between TRBV27 relative expression and the onset of acute graft-versus-host disease. Research carried out by Wang et al^27^ reported that a significant skewing of the TCR-BV repertoire occurred at the maternal–fetal interface when patients experienced a spontaneous abortion. Thus, skewing of the TCR-BV repertoire might be associated with susceptibility to unexplained pregnancy loss.

In this present study, we analyzed the degree of TCR-BV/J/D segments usage. TRBV20–1, TRBV2, TRBV19, TRBV5–1, and TRBV7–9 showed higher usage, whereas TRBV7–1, TRBV23–1, TRBV16, TRBV21–1, and TRBV6–7 exhibited significantly lower usage. As for the TCR-BJ segments usage, TRBJ2–7, TRBJ2–1, and TRBJ2–3 were highly expressed in T cells from healthy donors (mean values >10%), while TRBJ2–4, TRBJ2–6, and TRBJ1–3 were poorly expressed (mean values <3%). As for TCR-BD segments usage, the mean usage frequencies of TRBD1 and TRBD2 were 48% and 52%, respectively. Currently, it is difficult to explain the different degrees of TCR-B (V/D/J) repertoire usage. Notably, Connelley et al^28^ demonstrated that expansion of the genomic TRBV repertoire can occur via a complex and extensive series of duplications that predominantly involve blocks of DNA that contain multiple genes. These duplication events can result in the massive expansion of several TRBV subgroups. Additionally, to understand the evolutionary dynamics of the VB gene family, Su et al^29^ constructed phylogenetic trees of TCR-VB genes from humans and mice to examine changes in gene arrangements of the TCR-VB region throughout evolution. They found that the VB gene family had evolved by a birth-and-death process rather than by concerted evolution. Moreover, they presented evidence that a 20-kb VB region had duplicated in tandem 4 times in the human lineage during the last 32 Myr, and 6 of 15 VB genes in this region had become nonfunctional during this period.

The most striking finding from our study was that the usage frequency of individual nucleotides and amino acids within CDR3 intervals was remarkably consistent between individuals. The TCR CDR3 protein is known to be 1 of the most diversified and complex proteins in humans. We could observe some conserved features in the composition of CDR3. These intriguing findings clearly demonstrate that our understanding of the TCR repertoire remains incomplete, and remind us that exciting new rearrangement mechanisms still await discovery. Additionally, we found that Tdt was biased toward the insertion of G and C over A and T bases, in accordance with the results of a previous study that also described a similar Tdt mononucleotide insertion bias.^9^ In summary, high-throughput sequencing of TCR sequences offers many opportunities to gain new insights into the human adaptive immune system. By applying the method described herein, we identified some TCR-β repertoire features that may inform future studies of human TCR gene recombination.

## Supplementary Material

Supplemental Digital Content

## Supplementary Material

Supplemental Digital Content

## Supplementary Material

Supplemental Digital Content

## Supplementary Material

Supplemental Digital Content

## Supplementary Material

Supplemental Digital Content

## Supplementary Material

Supplemental Digital Content

## Supplementary Material

Supplemental Digital Content

## Supplementary Material

Supplemental Digital Content

## Supplementary Material

Supplemental Digital Content

## Supplementary Material

Supplemental Digital Content

## Supplementary Material

Supplemental Digital Content

## Supplementary Material

Supplemental Digital Content

## Supplementary Material

Supplemental Digital Content

## Supplementary Material

Supplemental Digital Content

## Supplementary Material

Supplemental Digital Content

## Supplementary Material

Supplemental Digital Content

## Supplementary Material

Supplemental Digital Content

## Supplementary Material

Supplemental Digital Content

## Supplementary Material

Supplemental Digital Content

## Supplementary Material

Supplemental Digital Content

## Supplementary Material

Supplemental Digital Content

## Supplementary Material

Supplemental Digital Content

## Supplementary Material

Supplemental Digital Content

## Supplementary Material

Supplemental Digital Content

## Supplementary Material

Supplemental Digital Content

## Supplementary Material

Supplemental Digital Content

## Supplementary Material

Supplemental Digital Content

## Supplementary Material

Supplemental Digital Content

## Supplementary Material

Supplemental Digital Content

## Supplementary Material

Supplemental Digital Content

## Supplementary Material

Supplemental Digital Content

## Supplementary Material

Supplemental Digital Content
