# Supplementary material for: High Throughput Sequencing of T Cell Antigen Receptors Reveals a Conserved TCR Repertoire
Source: Medicine (Baltimore). 2016 Mar 11;95(10):e2839. doi: 10.1097/MD.0000000000002839 (PMC4998859; doi:10.1097/MD.0000000000002839)
Supplement: Supplemental Digital Content [file medi-95-e02839-s022.docx]

Supplemental Table 1. A summary of highly expanded clones in each sample.

| Sample | Clonotype | Frequency (%) |
| --- | --- | --- |
| NC-1 | TGCAGCGCCCTACGACTAGCGGGGTACAATGAGCAGTTCTTC | 10.84 |
|  | TGCAGCGTTCGGACAGGGGGCGAGAGCTACACCTTC | 2.12 |
|  | TGTGCCAGCAGTCTCCCCGGGACATACGACTACGAGCAGTACTTC | 1.75 |
|  | TGTGCCACCAGTGGTATGTCAAATGAGCAGTTCTTC | 1.66 |
|  | TGCGCCAGCAGCCAAGAGGCGGTTAGCGAGCAATGAGCAGTTCTTC | 1.66 |
|  | TGTGCCAGCAGAAGGACAGGGTCGCCCAATCAGCCCCAGCATTTT | 1.48 |
|  | TGTGCCATCAGTGAGAAGAACAATCAGCCCCAGCATTTT | 1.39 |
|  | TGTGCCAGCAGCTTATCTGAACCGGGACCTGGGCTCCTACGAGCAGTACTTC | 0.91 |
|  | TGTGCCACCAGCAGAGAGATTTGGGACAACTACGAGCAGTACTTC | 0.84 |
|  | TGTGCTACGAGGGACCCACCCACAGATACGCAGTATTTT | 0.82 |
|  | TGTGCCAGCAGCTCTCCGGTCGGGTTTATGAACACTGAAGCTTTCTTT | 0.77 |
|  | TGTGCCAGCAGCCAGGACAGGTTAGCTAACTATGGCTACACCTTC | 0.77 |
|  | TGCGCCAGCAGCCGGTCACTAGCGGCACTCCTACGAGCAGTACTTC | 0.64 |
|  | TGTGCCACCAGTGGATACGGGAATGAGCAGTTCTTC | 0.49 |
|  | TGCAGTGCTATGGGGTACTACTACTAGCGGGAGTCGGAACGAGCAGTACTTC | 0.43 |
|  | TGTGCCATCAGTGAGGACGGATGGAACGAGCAGTACTTC | 0.41 |
|  | TGCAGTGCTGGAAGGACTAGCGGGATTCGAGAGACCCAGTACTTC | 0.38 |
|  | TGTGCCATCAGTGAGGAAGTTCCAGATACGCAGTATTTT | 0.35 |
|  | TGTGCCAGCAGCTACGCAGTGAACACTGAAGCTTTCTTT | 0.26 |
|  | TGTGCCAGCAGTGTCGAGTACGGGGAGCTGTTTTTT | 0.26 |
|  | TGTGCCAGCAGTCAGGGCGCCAATCAGCCCCAGCATTTT | 0.23 |
|  | TGTGCCAGTAGCCACGGACAGTTCTACGAGCAGTACTTC | 0.23 |
|  | TGCAGTGCTAGAGGATCGTCTAGCGACTACGAGCAGTACTTC | 0.21 |
|  | TGCAGCGTTGAGCGGCACTGAAGCTTTCTTT | 0.20 |
|  | TGTGCCACCACTGGCGGGAGCGGGCAATGAGCAGTTCTTC | 0.20 |
|  | TGCAGCGCCCAGGGAAACTCAGAACGGGATACAATGAGCAGTTCTTC | 0.19 |
|  | TGCGCCAGCAGCCAAGATCCGTCAGGCTCTGGCTACACCTTC | 0.19 |
|  | TGTGCCACCAGTGGCCTGGCCAATGAGCAGTTCTTC | 0.19 |
|  | TGTGCCACCAGCAGAGTGTCAGACCAAGAGACCCAGTACTTC | 0.18 |
|  | TGTGCCAGTCGACAGGGGGGATACAATGAGCAGTTCTTC | 0.18 |
|  | TGTGCCAGCAGCTTCCGACTAACAGATACGCAGTATTTT | 0.17 |
|  | TGTGCCACCAGCAGAGGGCTAGTAGTTGGACGAGCAGTACTTC | 0.16 |
|  | TGTGCCACCAGTGGCTACAGCAGTGAGCAGTTCTTC | 0.15 |
|  | TGCAGTGCTAGAGAGGCAGTCATGGAGTACGGGGAGCTGTTTTTT | 0.15 |
|  | TGTGCTTCGAGTGGAGGGGCATGGCAGCCCCAGCATTTT | 0.14 |
|  | TGTGCCAGCATGAGTAGCGGGGACACCGGGGAGCTGTTTTTT | 0.14 |
|  | TGCAGTGCTAGTCCGACCTGGGACTGAAGCTTTCTTT | 0.14 |
|  | TGTGCCTCCACCGACAGCTCCTATAATTCACCCCTCCACTTT | 0.13 |
|  | TGTGCCAGCAGCCTACGGGACTCCCTAGCGGAACATAGCACAGATACGCAGTATTTT | 0.13 |
|  | TGTGCCAGCAGTTCTAGCGGGAGGGCGGCAATGAGCAGTTCTTC | 0.13 |
|  | TGTGCCAGCAGAGGACTAGCGGGAGCAGTTCTTC | 0.13 |
|  | TGCGCCAGCAGTGACTAGCGGGAGCAATGAGCAGTTCTTC | 0.13 |
|  | TGCAGCGCTCAGAGGGTAGACAGAACCCCGGACACTGAAGCTTTCTTT | 0.13 |
|  | TGTGCTAGCAGCTTAGCGTCCCCCAGACTAGCTAAGGGTTTACGAGCAGTACTTC | 0.13 |
|  | TGTGCCAGCAGCTTAAGGGCTAGCGGGATTTGGGATACGCAGTATTTT | 0.13 |
|  | TGTGCCAGCAGTCCAGGGGGAGTGAACTATGGCTACACCTTC | 0.12 |
|  | TGTGCCACCAGTGGGGGGAGCAATGAGCAGTTCTTC | 0.12 |
|  | TGTGCCAGTGAGGTCGACTAGCGGGGTTACACAGATACGCAGTATTTT | 0.12 |
|  | TGTGCCACCAGCCCTATGACTAGCGGGAGCGAGACCCAGTACTTC | 0.11 |
|  | TGTGCCAGCAGCGGACAGACAGGGGCGAGTTCACCCCTCCACTTT | 0.11 |
|  | TGTGCCAGCAAGGTCTCTGGGACCGCCGGGGAGCTGTTTTTT | 0.11 |
|  | TGTGCCACCAGCACTAAGTCAGCGGGAGCAACCGAGGACAGCACAGATACGCAGTATTTT | 0.10 |
|  | TGTGCCAGTAAAGTTGACGGGATGGATGGGGAGCTGTTTTTT | 0.10 |
| NC-2 | TGTGCCAGTACTAAGGGGGTTGGATTAGAGACCCAGTACTTC | 13.01 |
|  | TGTGCCAGTAGTATCTCCCAAGACACTGAAGCTTTCTTT | 3.79 |
|  | TGTGCCAGTAGTATATCTCAGGACACTGAAGCTTTCTTT | 3.45 |
|  | TGTGCCAGTAGTATAAGTCAAGACACTGAAGCTTTCTTT | 2.99 |
|  | TGCAGTGCCATAAAACCGGGACTAGGGCTACGAGCAGTACTTC | 2.41 |
|  | TGTGCCAGCAGCCACAACAGGGGGATACAAACTGAAGCTTTCTTT | 1.71 |
|  | TGTGCCAGCAGTCCAGGGACAGGGCCCTACGAGCAGTACTTC | 1.53 |
|  | TGTGCCAGCAGCCCAACAGGGGGCACTGAAGCTTTCTTT | 1.37 |
|  | TGTGCCAGCAGCTCGACAGGGGTGGATTCGGATACGCAGTATTTT | 1.34 |
|  | TGCAGCGTGAGGGCTGGCCAAGAGACCCAGTACTTC | 1.30 |
|  | TGTGCCAGCAGCTCCTCAGAGGGGGACAGAGCTAACGTTTATGGCTACACCTTC | 1.26 |
|  | TGCAGTGCTTATTACCGCCCCAGCTTCAACTACGAGCAGTACTTC | 1.24 |
|  | TGTGCCAGCAGCTTGGTGTTGTCGGGGGAGCAGTTCTTC | 0.88 |
|  | TGTGCCAGCAGCCCCACGAGCAGTTCGAACTATGGCTACACCTTC | 0.63 |
|  | TGCAGTGCTATTTCCGTCGACGCCCCCTACGAGCAGTACTTC | 0.47 |
|  | TGTGCCAGCAGCTTGGGGTTAGCGGGAGACAATGAGCAGTTCTTC | 0.43 |
|  | TGTGCCAGCAGCTTAGTACAGGTGGGGAGTGTTAGCAGTTCTTC | 0.41 |
|  | TGTGCCACCAGCGGCCTCGGGGATACGCAGTATTTT | 0.41 |
|  | TGTGCCAGCAGTGAGGCTCCGTCTGGGGACACTGAAGCTTTCTTT | 0.36 |
|  | TGTGCCAGCAGCTGGGACTTCTACAATGAGCAGTTCTTC | 0.36 |
|  | TGTGCCAGCAGCTTCTAACAAGAACCGGGACCGCGAACACTGAAGCTTTCTTT | 0.34 |
|  | TGTGCCAGCAGTTACCCCACGGCGGAGGGGGAGGAGACCCAGTACTTC | 0.27 |
|  | TGTGCCACCACGGGAGGGGAATACAATGAGCAGTTCTTC | 0.27 |
|  | TGTGCCAGCAGCCTCCTAAGGGGGGAGAACACTGAAGCTTTCTTT | 0.25 |
|  | TGTGCCATGTCGTACGGAAAAACTGTTTTTT | 0.25 |
|  | TGTGCCAGCAGTTACGGTCGACAGGATCCTCACCCGATTGGATATGGCTACACCTTC | 0.24 |
|  | TGCAGCGGTGAAAAGGGGACAGGGGTCAATGAGCAGTTCTTC | 0.23 |
|  | TGCAGTGCTAGAGATTATAGGGGGCGTACAGATACGCAGTATTTT | 0.23 |
|  | TGTGCCAGCAGTCACTCGGCTGAGCAGTACTTC | 0.19 |
|  | TGTGCCAGCAGCTTGCCCCCGATTACCTACGAGCAGTACTTC | 0.17 |
|  | TGTGCCAGCAGTGAAGATTTCAGGGGGGACTATGGCTACACCTTC | 0.17 |
|  | TGCAGTGCTAGGAGGACGGATAATCAGCCCCAGCATTTT | 0.16 |
|  | TGTGCCATCAGTGAGACAGGAACTAATGAAAAACTGTTTTTT | 0.15 |
|  | TGTGCCAGCAGCCCAGACCTGGGGGGCAATCAGCCCCAGCATTTT | 0.15 |
|  | TGTGCCAGCAGCCCGGGCCGCCAGCCCCAGCATTTT | 0.13 |
|  | TGTGCCAGCAGCCAAGGTGTCGGCTCCTACGAGCAGTACTTC | 0.13 |
|  | TGTGCCACCAGTGATTCGCAAGGACCACTGTCGGCAGACTACGAGCAGTACTTC | 0.13 |
|  | TGCAGTGCTTGGACGGGCGCTCAGGGTGAGCAGTTCTTC | 0.12 |
|  | TGTGCCAGCAGTTACAAGACTAGGCGCAATGAGCAGTTCTTC | 0.12 |
|  | TGCAGCGTCTACGACAAGGGGATAGCGGCAGATACGCAGTATTTT | 0.11 |
|  | TGTGCCAGCAGTCTGGTGTACAATGAGCAGTTCTTC | 0.11 |
|  | TGTGCCAGTAGTTCGACTAGCGGGCCCTACGAGCAGTACTTC | 0.10 |
|  | TGTGCCAGCAGCGTAGGGGGGAGGGGGACCGAAGCTTTCTTT | 0.10 |
| NC-3 | TGCAGTGCTCCTAGAGCAGGCATAGCTGACAATGAGCAGTTCTTC | 3.46 |
|  | TGTGCCAGCAGACCCCCGGGCGCTGGAAACACCATATATTTT | 1.33 |
|  | TGTGCCAGCAGTCAGACAGGGCCCTGGGGTGGCTACACCTTC | 0.34 |
|  | TGTGCCAGCAGGGGGACAGGGGGTAATCAGCCCCAGCATTTT | 0.30 |
|  | TGCGCCAGCAGCTTGAGGACCGGAGTGTATTCTGAAGCTTTCTTT | 0.24 |
|  | TGTGCCAGCACCTGGGAGAGGGGACGAACTGGCTACACCTTC | 0.18 |
|  | TGTGCCAGTAGTCCATTATGGACAGGCCGAACTATGGCTACACCTTC | 0.13 |
|  | TGTGCCAGCAGTTACTACCACTGGGGGACAGGGGTCAATCAGCCCCAGCATTTT | 0.12 |
|  | TGCGCCAGCAGTGATACAGGGAGTATCTACGAGCAGTACTTC | 0.12 |
|  | TGTGCCAGCAGCTACGTGGGACGGCTGAACACTGAAGCTTTCTTT | 0.12 |
|  | TGTGCCAGCAGTCCAGCGGATTCGAACACCGGGGAGCTGTTTTTT | 0.11 |
|  | TGTGCCAGCAGCTTCCAAACATGGGAAAGGGCTAATGAAAAACTGTTTTTT | 0.11 |
|  | TGTGCCAGCAGCTTAACGTCAGGCGGAGGAACACTGAAGCTTTCTTT | 0.10 |
|  | TGTGCCAGCAGCTTAGCGGCCGGCAGCTCTGGAAACACCATATATTTT | 0.10 |
| NC-4 | TGTGCCAGCAGCTTAGCGGGGGGGACCCCACCTATCTACGAGCAGTACTTC | 15.04 |
|  | TGTGCCAGCAGCTTATTATCGGCGGGACTCCTACGAGCAGTACTTC | 5.26 |
|  | TGCAGCCCGAGGAGGAGCAGTTCTTC | 3.88 |
|  | TGCAGTGCTGCGGGGGGGACAGACCATCTTTCTGAGCAGTACTTC | 3.80 |
|  | TGTGCCAGCAGCGGGTCGACAGCTGGGAGCTACGAGCAGTACTTC | 0.76 |
|  | TGTGCCAGCAAGAAACCGGGACAAACAACTAATGAAAAACTGTTTTTT | 0.76 |
|  | TGTGCCAGCAGTGATCCCGGTGCCGGGCGAGAGACCCAGTACTTC | 0.70 |
|  | TGTGCCAGCAGCTTAGCGCTAACTAGGGGGGATGAGCAGTTCTTC | 0.48 |
|  | TGTGCCAGCAGCGCGCCCAATTCACCCCTCCACTTT | 0.41 |
|  | TGTGCCAGTAGTATAGGGGGGACACGCTCAGTCGGCAATCAGCCCCAGCATTTT | 0.31 |
|  | TGCAGCGTTGAAAATCAGGGGAACTATGGCTACACCTTC | 0.20 |
|  | TGTGCCAGCAGTTACGAGCTATACAATGAGCAGTTCTTC | 0.18 |
|  | TGTGCCAGCAGCCCAGGGGTAGGGACTAATGAAAAACTGTTTTTT | 0.17 |
|  | TGTGCCAGCAGCCTCCGGGACAGGGCCATCAATGAGCAGTTCTTC | 0.17 |
|  | TGTGCCAGCAGCTTAGCGGGGGGACCCCACCTATCTACGAGCAGTACTTC | 0.15 |
|  | TGCAGTGCTAGAGATGAGACAGTTTCTAACTATGGCTACACCTTC | 0.11 |
|  | TGTGCCAGCAGCAACAATGAGCAGTTCTTC | 0.11 |
| NC-5 | TGCAGCGTTGGACTCAATGGGCGCGAGCAGTACTTC | 17.23 |
|  | TGTGCCACCAGTGATGGCGACTGACAGGAGGGCTACGAGCAGTACTTC | 4.58 |
|  | TGCGCCAGCAGTGAGTCGGACTCTGGGGCCAACGTCCTGACTTTC | 1.78 |
|  | TGTGCCAGCAGTTACTCGGGGGCGGACGAGCAGTACTTC | 0.94 |
|  | TGTGCCACCAGTGATTTGCCGGATCCACTTT | 0.85 |
|  | TGTGCCAGCAGCTTAGAGGCCGTTTCCTACAATGAGCAGTTCTTC | 0.56 |
|  | TGTGCCAGCAGTGAAGACCCCCGGCCCTCGACTGCGGATGGCTACACCTTC | 0.56 |
|  | TGTGCCAGCAGCGCCCACGGGGCGGGGGCCAACGTCCTGACTTTC | 0.56 |
|  | TGTGCCAGCAGTTACTCGGGCTCCGACGAGCAGTACTTC | 0.54 |
|  | TGTGCCAGCAGTCAATTTTAGGACCCTGGACTAGCGGTTGAGCAGTTCTTC | 0.54 |
|  | TGTGCCAGCAGTTTAAGACAGGGAGAACAGCCCCAGCATTTT | 0.44 |
|  | TGTGCCAGCTTGGCCCCCTACGAGCAGTACTTC | 0.44 |
|  | TGTGCCAGCAGCTTAGGGGGAGATGGCTACACCTTC | 0.41 |
|  | TGTGCCACCAGCAGAGATTCTACAGGGGGCGGGTATGGCTACACCTTC | 0.40 |
|  | TGCAGTGCTAGATCTGGGAGGGCCTCGAGGGAGCAGTACTTC | 0.39 |
|  | TGTGCCATCAGTGTGTCAGGAAATAGCAATCAGCCCCAGCATTTT | 0.37 |
|  | TGTGCCAGCAGCTTAAGATCGGACAGAACTAGCGGGCCGGAGGCGGCAGATACGCAGTATTTT | 0.36 |
|  | TGTGCCAGCAGAAATGCGCTTCTCTGGATGAACACTGAAGCTTTCTTT | 0.36 |
|  | TGTGCCAGCAGCGTAGGAAACGGGGGGGGTCAGGAGACCCAGTACTTC | 0.31 |
|  | TGTGCCAGCAGCTCTGGGACAGGGGTACCTCCTACGAGCAGTACTTC | 0.28 |
|  | TGTGCCAGCAGTGGGGGGACAGGGACTTTTGAGACCCAGTACTTC | 0.28 |
|  | TGCGCCAGCAGTGAGCAGGGAATGAGCAGTTCTTC | 0.27 |
|  | TGTGCCATCAGTGTCTCCGGAAATAGCAATCAGCCCCAGCATTTT | 0.25 |
|  | TGCGCCAGCAGCCAAGATGTCGCAGGGGTGGCGAACACTGAAGCTTTCTTT | 0.25 |
|  | TGTGCCAGAGAGAGAGGGACGGATGAACACTGAAGCTTTCTTT | 0.24 |
|  | TGCGCCAGCAGTGGAGGGCAGGGGGGTACGAGCAGTACTTC | 0.24 |
|  | TGTGCCAGCAGCGGAAAATACACTGAAGCTTTCTTT | 0.24 |
|  | TGTGCCAGCAGCGTAGGACAGGGAGGGCCTGAGTTCTTC | 0.23 |
|  | TGCAGCGCGGAAGGGACTAGCGGGAGTGGGACCGGGGAGCTGTTTTTT | 0.23 |
|  | TGTGCCAGCAGTTTGGGGTTCCAGGGCGAAGCTTTCTTT | 0.21 |
|  | TGTGCCAGCAGTCCAGGGGCTGGCTACACCTTC | 0.21 |
|  | TGTGCCAGCTTCAGGGTACCCCGTCAAGTGCAGTTCTTC | 0.20 |
|  | TGTGCCACCAGTGATCTACAGGGGAGTGATGGCTACACCTTC | 0.19 |
|  | TGTGCCAGCATCACATCCTCCCTGGTACCTCTGGGGCCAACGTCCTGACTTTC | 0.19 |
|  | TGTGCCAGCAGCTTAAATTCTGGCGGGGTAAGCTCCTACGAGCAGTACTTC | 0.15 |
|  | TGTGCCTGGAGTGGACGGGGGCCCCAAGAGACCCAGTACTTC | 0.15 |
|  | TGTGCCAGCGTCCTGGATACAGGGTTCGATGAAAAACTGTTTTTT | 0.15 |
|  | TGTGCCAGCAGCGTAGACCCCCGCTACGACTGGAAACACCATATATTTT | 0.15 |
|  | TGTGCCAGCAGCTTGGACGTCACAGATACGCAGTATTTT | 0.14 |
|  | TGTGCCAGCAGTGAATATGGCATGAACACTGAAGCTTTCTTT | 0.13 |
|  | TGTGCCAGCAGCTACAGGGTTGCAATCAGCCCCAGCATTTT | 0.13 |
|  | TGCAGCGTCTTAGAGGACTATGGCTACACCTTC | 0.13 |
|  | TGTGCCAGCAGCGTAGCTTTGGTGAGGCTACACCTTC | 0.13 |
|  | TGTGCCAGCAGCTTAGGTGGGGGCCCAACTAATGAAAAACTGTTTTTT | 0.13 |
|  | TGTGCCAGCAGTTATGTATTGGGGCGAGCTCCTATAATTCACCCCTCCACTTT | 0.12 |
|  | TGCGCCAGCAGCTATACAGGGAACTTT | 0.12 |
|  | TGTGCCAGCAGCGGCGCAACCTATAATTCACCCCTCCACTTT | 0.12 |
|  | TGTGCCAGTAAAGTTTCACAGGCCTCAGGAAATGAAAAACTGTTTTTT | 0.11 |
|  | TGTGCCAGCAGTGGGGAGTTCGGATTCACCCCTCCACTTT | 0.10 |
|  | TGTGCCAGTAGTCTCGACAGGGGCGGATACGAGCAGTACTTC | 0.10 |
| NC-6 | TGTGCCACCAGCAGAGATACGGCAGGCACTGAAGCTTTCTTT | 2.63 |
|  | TGCAGTGCTAGAGGGGACTCGGGGATCTACGAGCAGTACTTC | 0.90 |
|  | TGCAGTGTCCGGGGACAGGCCTACGAGCAGTACTTC | 0.71 |
|  | TGCGCCAGCAGCTTGGCTAGGGCAGGGGGCGCGTCTGGCTACACCTTC | 0.52 |
|  | TGTGCCAGCAGCGTAGAGCGGAGCGGCCAGGGGCTGTTCTTC | 0.47 |
|  | TGTGCCAGTAGTCCTCCCGCAGACTACTATGGCTACACCTTC | 0.46 |
|  | TGTGCCACCAGCAACCCTAGCGGAACAAATAAATACGAGCAGTACTTC | 0.41 |
|  | TGTGCCAGCAGTTCTCGTCTAGCGGGAGGTACTGACGAGCAGTACTTC | 0.38 |
|  | TGTGCCAGCAGTGAAACAGGGTCCAGCACAGATACGCAGTATTTT | 0.37 |
|  | TGTGCCAGCAGTACCCTTAGCGGGGGGGGGCAATGAGCAGTTCTTC | 0.35 |
|  | TGCGCCAGCAGCCCTATGGACAGCCAGTTCTACGAGCAGTACTTC | 0.30 |
|  | TGTGCCAGCAGCTCGGGGGCAGAGCAGTTCTTC | 0.29 |
|  | TGTGCCACCAGGCCCTTAGACTCGAATACGCAGTATTTT | 0.27 |
|  | TGTGCCAGCACCTTCCTGAGGCTAGCGGGGTGCAATACGAGCAGTACTTC | 0.26 |
|  | TGCAGCGTAAGAGCGGGAGGGAATGAGCAGTTCTTC | 0.24 |
|  | TGTGCCAGCAGGGACTCCGGACAGGCCTACGAGCAGTACTTC | 0.23 |
|  | TGTGCCAGCAGCTTGGGGGGACGGGGCAGCAATCAGCCCCAGCATTTT | 0.23 |
|  | TGTGCCAGCAGTGAGACGGGTTCGAGCACAGATACGCAGTATTTT | 0.22 |
|  | TGTGCCAGCAGCCCCTCCGGGACAGGGTTTCCACCCCTCCACTTT | 0.20 |
|  | TGTGCCTGGAATCGGGGACAAGCATTCACCCCTCCACTTT | 0.19 |
|  | TGTGCCAGCAGCTTAGACAGGGAAACCTACGAGCAGTACTTC | 0.18 |
|  | TGTGCCAGCAGCTTAGATCCTAAAAAATAGCGGGAGTTCGCATCGGATACGCAGTATTTT | 0.18 |
|  | TGTGCCAGCAGCGAAACAGGGTCAGATGAAAAACTGTTTTTT | 0.16 |
|  | TGCAGCGTTGAAGTGGACACAGGGACTGAAGCTTTCTTT | 0.16 |
|  | TGTGCCAGCAGCGCGAGTCGGGAATCTCCCTGGTACAGATGAGCAGTTCTTC | 0.14 |
|  | TGTGCCAGCAGCTTACCCACCTCCGGGACCGATCGCTACGAGCAGTACTTC | 0.13 |
|  | TGTGCCAGCAGTAAAAGGGACAGGGTTTGTCCGGGTCAGCCCCAGCATTTT | 0.11 |
| NC-7 | TGTGCCAGTAGCCCTGCCAGCTCCTACAATGAGCAGTTCTTC | 0.90 |
|  | TGTGCCATCAGTGATCTGGGGGGTACCTACGAGCAGTACTTC | 0.80 |
|  | TGTGCCAGCAGCTTGGTAGGACAGGAGAACGAGCAGTACTTC | 0.59 |
|  | TGCGCCAGCAGTGAAGAGGGCTATAGCAATCAGCCCCAGCATTTT | 0.56 |
|  | TGTGCCAGCACCCGGGCGGACGTTGTCATCAAGGAGCACAGATACGCAGTATTTT | 0.53 |
|  | TGTGCCAGCAGTGACTCAGGGGAAACAGATACGCAGTATTTT | 0.41 |
|  | TGTGCCACCAGTGATTTAGACAATCAGCCCCAGCATTTT | 0.41 |
|  | TGCAGCGTCCGCGCTGGGGGGAACGAGCAGTACTTC | 0.40 |
|  | TGTGCCACCAGTGATTTTTCGGGGGACTTC | 0.39 |
|  | TGTGCCACCAGCAGAGACATACGGGACAGGACCGATAGACCTTC | 0.35 |
|  | TGCAGTCCTAGGGGGGGAGATGGAAATGAGCAGTTCTTC | 0.31 |
|  | TGCGCCAGCCGTAGCGGGAGGGCTCTCATAAGAGACCCAGTACTTC | 0.29 |
|  | TGTGCCAGCAGCGTAGAAGGGAGGGATACGCAGTATTTT | 0.28 |
|  | TGTGCCAGTACCCCATTCCTAGCGGGAGACAATGAGCAGTTCTTC | 0.27 |
|  | TGTGCCAGCAGCTTAGACAGGGGGCGTTACGAGCAGTACTTC | 0.26 |
|  | TGTGCCACCAGCATGTCACGGGAAGGCTACAATGAGCAGTTCTTC | 0.25 |
|  | TGCAGTGCTAGAGTCATTGCCTAGCGGGAGGATCCTACGAGCAGTACTTC | 0.24 |
|  | TGTGCCATCAGTGAGTCGGTCGACATACGAACACCGGGGAGCTGTTTTTT | 0.23 |
|  | TGCGCCAGCAGTGATCAGGCGGAGGGGGATCAGCCCCAGCATTTT | 0.23 |
|  | TGCAGTGCTATATTCACAGCTAGCTACGAGCAGTACTTC | 0.21 |
|  | TGTGCCAGCAGCTCCCTGTCACAAGGGGACTACGAGCAGTACTTC | 0.20 |
|  | TGTGCCAGCAGTAGCGGACAGGAAGTCACAGATACGCAGTATTTT | 0.19 |
|  | TGTGCCAGCAGTGATTTAACAGGGGCGGGTGAAAAACTGTTTTTT | 0.19 |
|  | TGTGCCAGCAGTGAATCGATGGAGCGGGAGACCCAGTACTTC | 0.19 |
|  | TGTGCCAGCAGCGTAGACGACCGGCGTGGATCCCACAATGAGCAGTTCTTC | 0.18 |
|  | TGTGCCAGCAGCTTAGGACTAGCGGCGCTCACCGGGGAGCTGTTTTTT | 0.16 |
|  | TGCGCCAGCAGCCACGAACAGGGGGCTCGGGCAGATACGCAGTATTTT | 0.15 |
|  | TGCAGCGTTGCATGACCCCATTCCGCTACAATGAGCAGTTCTTC | 0.15 |
|  | TGTGCCAGCAGCTCCCGATGACTAGCGGGAACCCCTAGGGCAGTACTTC | 0.15 |
|  | TGTGCCAGCAGTGCGACAGGTTACTACGAGCAGTACTTC | 0.14 |
|  | TGTGCCAGCAGCCTAGACAGGGAATCAGCCCCAGCATTTT | 0.13 |
|  | TGTGCCAGCAGCTTAGCGATTTCAGACAGGCCAAACACTGAAGCTTTCTTT | 0.13 |
|  | TGCAGTGCTAGAGTGGGACATCGAATTAAGACCCAGTACTTC | 0.13 |
|  | TGCAGCGTTGGCGGGAGTAGTACCGGGGAGCTGTTTTTT | 0.12 |
|  | TGCGCCAGCAGCTTGGTAGCGGGGCTACGAGCAGTACTTC | 0.12 |
|  | TGTGCCAGCAGGACAGGGGGCGAAAAACTGTTTTTT | 0.12 |
|  | TGCAGTGCACCGGGACAGAGGAGGCCAAAGGTCATTCAGTACTTC | 0.12 |
|  | TGCAGTGCTAGAGGACAGGGGGACACCGGGGAGCTGTTTTTT | 0.12 |
|  | TGCGCCAGCAGCCCAGGGGGGACAGCGCAGTATTTT | 0.12 |
|  | TGCGCCAGCAGCCCATGGACAGGGACCAACACTGAAGCTTTCTTT | 0.12 |
|  | TGCGCCAGCAGCTTGCAGGGTTCATCTGGCTACACCTTC | 0.12 |
|  | TGTGCCATCAGTGAGCTAAGTGGCGAGCAGTACTTC | 0.11 |
|  | TGCAGTGCTAGAGCCCGACTGATACAAGAGACCCAGTACTTC | 0.11 |
|  | TGTGCCAGCAGCTTAGGGACAGGACCCTACAATGAGCAGTTCTTC | 0.11 |
|  | TGTGCCAGCAGCGTAGGGACTAGCAATGAGCAGTTCTTC | 0.10 |
|  | TGCAGCGTTGAAGGGCAGGGAGGAAACACCATATATTTT | 0.10 |
|  | TGTGCCAGCAGCCCAGTTCTAGCGGGAGGAGTGACAGATACGCAGTATTTT | 0.10 |
|  | TGTGCCAGCAGCTTAGGACGGATTGGGGGACGGTACGAGCAGTACTTC | 0.10 |
| NC-8 | TGTGCCAGCAGCTCGCAGAACTATGGCTACACCTTC | 23.27 |
|  | TGTGCCAGCACAGACAGGGACTCTCCTACGAGCAGTACTTC | 4.36 |
|  | TGCGCCAGCAGCTTGGCGGGGACAGGGGTGATTCACCCCTCCACTTT | 2.93 |
|  | TGTGCCAGCAGCCCCGGGGGGGGGACCTACGAGCAGTACTTC | 1.00 |
|  | TGTGCCAGCAGCGCCATCCTCCTACGAGCAGTACTTC | 0.84 |
|  | TGTGCCAGTAGTATGGCGGGCAATGAGCAGTTCTTC | 0.63 |
|  | TGTGCCAGCAGCCAAGATGACGGAACTGGAAACACCATATATTTT | 0.62 |
|  | TGTGCCAGCAGCGACGAGGGCACCCACTATGGCTACACCTTC | 0.50 |
|  | TGCAGTGCTAGAGACACCTTTCCCTACGAGCAGTACTTC | 0.41 |
|  | TGTGCCAGTAGTCCCCCGGGCGAGCAGTACTTC | 0.41 |
|  | TGCAGTGCTAGCCCGAGCTCCTACGAGCAGTACTTC | 0.40 |
|  | TGCAGTGCTAGTCCTCAGTCCTACGAGCAGTACTTC | 0.34 |
|  | TGCGCCAGCAGCTTGGGACAGGGCGAAGCTTTCTTT | 0.33 |
|  | TGTGCCACCAGCAGAGATGGGACACCGGGACAGGGGGATTCGGCAGTACTTC | 0.29 |
|  | TGTGCCAGCAGTCGTCGGGACAGGGCCCCGGGGAGCAGTACTTC | 0.26 |
|  | TGTGCCAGCAGTGAACAAGAGACCCAGTACTTC | 0.24 |
|  | TGCGCCAGCAGCTTGTACGGACTAGCGGGAGGAGAGACCCAGTACTTC | 0.23 |
|  | TGTGCCAGCCTGGGAAACACTGAAGCTTTCTTT | 0.18 |
|  | TGTGCCAGCAGCCCCGGGGGGGGCCTGGGGAGAGAGCCCCAGCATTTT | 0.18 |
|  | TGTGCCAGTAGTATAGGCGGGGAGCTGTTTTTT | 0.16 |
|  | TGCAGCGTCCAGGGGGCGCTTTACGAGCAGTACTTC | 0.16 |
|  | TGCAGTGCTAGAGATCTGAGCGGGGATTGGGTGCAAGAGACCCAGTACTTC | 0.16 |
|  | TGTGCCAGCAGCGCGACAGGGAGCACAGATACGCAGTATTTT | 0.15 |
|  | TGTGCCAGCAGTGAGAGACAGGGGGCAGAATCAGCCCCAGCATTTT | 0.15 |
|  | TGTGCCATCAGTGGTGAACAGCGGTCCTACGAGCAGTACTTC | 0.13 |
|  | TGTGCCAGCAGTGGTAGACAGGGGGTGGTAGGTGAAAAACTGTTTTTT | 0.13 |
|  | TGTGCCAGTACTCCCGGACTAGCGGGGGAGAGACCCAGTACTTC | 0.13 |
|  | TGTGCCAGCAGCTCAAACCGGGACCTAGAGCGAGCAGTACTTC | 0.12 |
|  | TGCAGTGCTAGAGTACCGACAACCCCCGCACACCCCTCCACTTT | 0.12 |
|  | TGTGCCAGCAGTGACTCGAAGGTTGCGGGATTGGAGCAAGAGACCCAGTACTTC | 0.12 |
|  | TGTGCCAGCAGCTTATCACTGAGCTCCTACGAGCAGTACTTC | 0.11 |
|  | TGCAGCGTTGTCACGGTGAGGGACAGGGATGGGATACGCAGTATTTT | 0.11 |
|  | TGCGCCAGCAGCTTGGATAGGGGGTAGCAATCAGCCCCAGCATTTT | 0.11 |
|  | TGCAGTGCTAGAGAAACCCCAGCGGGGGATGAGACCCAGTACTTC | 0.11 |
|  | TGCAGTGCTAGGACTAAGGGGGGGGTTTCAGATACGCAGTATTTT | 0.11 |
|  | TGTGCCAGCAGTTTAAACGACAGGCTGAACACAGATACGCAGTATTTT | 0.10 |
|  | TGTGCCAGCAGTTTAGAACAGGGGGTTGGTGAAAAACTGTTTTTT | 0.10 |
| NC-9 | TGTGCCAGCAGTGAGGGGACAGAGACCCAGTACTTC | 9.81 |
|  | TGTGCCACCAGTGATAGCGGCCATCGTGGGACCTACAATGAGCAGTTCTTC | 7.04 |
|  | TGTGCCTGGAGAGGCGATGAGCAGTTCTTC | 5.97 |
|  | TGTGCCAGTAGTCCCAGGAACTACAATGAGCAGTTCTTC | 1.78 |
|  | TGTGCCAGCAGCGGATCTAGAGAGGGCTCCTACGAGCAGTACTTC | 1.12 |
|  | TGCAGCGTTGACCCCACTTACACTGAAGCTTTCTTT | 1.09 |
|  | TGCAGCGTGGGCGTGGACAGCAACACTGAAGCTTTCTTT | 0.70 |
|  | TGTGCCAGCACCTCCTTTGGGGGAACGAGGGATACGCAGTATTTT | 0.65 |
|  | TGCAGCGTTGGTTCGGACAGCAACACTGAAGCTTTCTTT | 0.55 |
|  | TGTGCCAGCAGCCCTTTGGGCGTTAGCAATCAGCCCCAGCATTTT | 0.47 |
|  | TGTGCCACCAGTGATTTGCCGGGACCGCTACGAGACCCAGTACTTC | 0.34 |
|  | TGTGCCACCAGCAGAGGGGCGGGAGGGCCCTACGAGCAGTACTTC | 0.34 |
|  | TGTGCCAGCAGTTTGGGACAGAAGAATGAAAAACTGTTTTTT | 0.33 |
|  | TGTGCCAGCAGGAAAATAGACAGATACGCAGTATTTT | 0.30 |
|  | TGCGCCAGCAGCCAAGGGCCCGAGGGGGGGGGAGGGCTACACCTTC | 0.29 |
|  | TGTGCCAGCAGCGTGGGGGGTACGACTAATGAAAAACTGTTTTTT | 0.25 |
|  | TGTGCCAGTAGTATGAGGGTCAATGAGCAGTTCTTC | 0.24 |
|  | TGTGCCAGTAGTTATCGGGTAAATGAGCAGTTCTTC | 0.24 |
|  | TGTGCCAGTAGTCCCAGGGTAAATGAGCAGTTCTTC | 0.22 |
|  | TGTGCCAGCAGTTTACCTATCGCAGGGAACTATGGCTACACCTTC | 0.22 |
|  | TGTGCCAGCAGTTTGAACCGGGACTAATTCTTATTCCGAACACCGGGGAGCTGTTTTTT | 0.16 |
|  | TGTGCCACCACTCGGACAGGGGCGAACACCGGGGAGCTGTTTTTT | 0.14 |
|  | TGTGCCAGCAGCTATGACAGACAGACCCAGTACTTC | 0.13 |
|  | TGTGCCAGCAGCTCAACCGTGGGAACCCTCGGACTAGCGGGAGTTAGTGGGTACGAGCAGTACTTC | 0.12 |
|  | TGTGCCAGCAGTTGGACAGGGTACGAGCAGTACTTC | 0.11 |
|  | TGCGCCAGCAAGTGGTCGACAGTCAATCAGCCCCAGCATTTT | 0.11 |
| NC-10 | TGTGCCAGTAGTACCGGGGGGGAAGCTCAGCCCCAGCATTTT | 1.22 |
|  | TGCAGCGTTGAAGGTTCCCGCCGGGACAGGCAGTTCTTC | 0.48 |
|  | TGTGCCAGCAGTGAGGGAAGATGGGGCTACGAGCAGTACTTC | 0.39 |
|  | TGTGCCAGTAGTATTGAGGAAAAACTGTTTTTT | 0.36 |
|  | TGTGCCAGCAGTGCTGGTGGGAGAGGATACGAGCAGTACTTC | 0.32 |
|  | TGCGCCAGCAGCTCTGAAGGGAACGAGCAGTACTTC | 0.31 |
|  | TGTGCCAGCAGTGACTCTAGCGGGAGCACAGATACGCAGTATTTT | 0.31 |
|  | TGTGCCAGCAGTCCGACAGGGGGCGGTCAGCCCCAGCATTTT | 0.25 |
|  | TGTGCCAGCAGCTTAGCGAGCACTGAAGCTTTCTTT | 0.23 |
|  | TGTGCCACCAGTGATTGACAGCACAGATACGCAGTATTTT | 0.21 |
|  | TGTGCCAGCAGTGAATCGGGGTTTTCGGATGGCTACACCTTC | 0.20 |
|  | TGTGCCAGCAGCCAAATTTCCAGCTCCTACAATGAGCAGTTCTTC | 0.17 |
|  | TGTGCCAGCACGGGGGGCTCTGGAAACACCATATATTTT | 0.16 |
|  | TGTGCCAGCAGTTCTTATGGCCTAACAGTAGGGGCAGTACTTC | 0.13 |
|  | TGTGCTAGTGATCGGGACAGGGGGACACCGGGGAGCTGTTTTTT | 0.13 |
|  | TGTGCCAGCAGCTTACACACACGACAGGGGACTTGATGCGAGCAGTACTTC | 0.12 |
|  | TGCGCCAGCAGCCAAGCACAGGGCCCTGGGAATGGCTACACCTTC | 0.11 |
|  | TGCGCCAGCAGTAACGGGACGGAGCACTACAATGAGCAGTTCTTC | 0.10 |
