# Supplementary figures and images for: High Throughput Sequencing of T Cell Antigen Receptors Reveals a Conserved TCR Repertoire
Source: Medicine (Baltimore). 2016 Mar 11;95(10):e2839. doi: 10.1097/MD.0000000000002839 (PMC4998859; doi:10.1097/MD.0000000000002839)

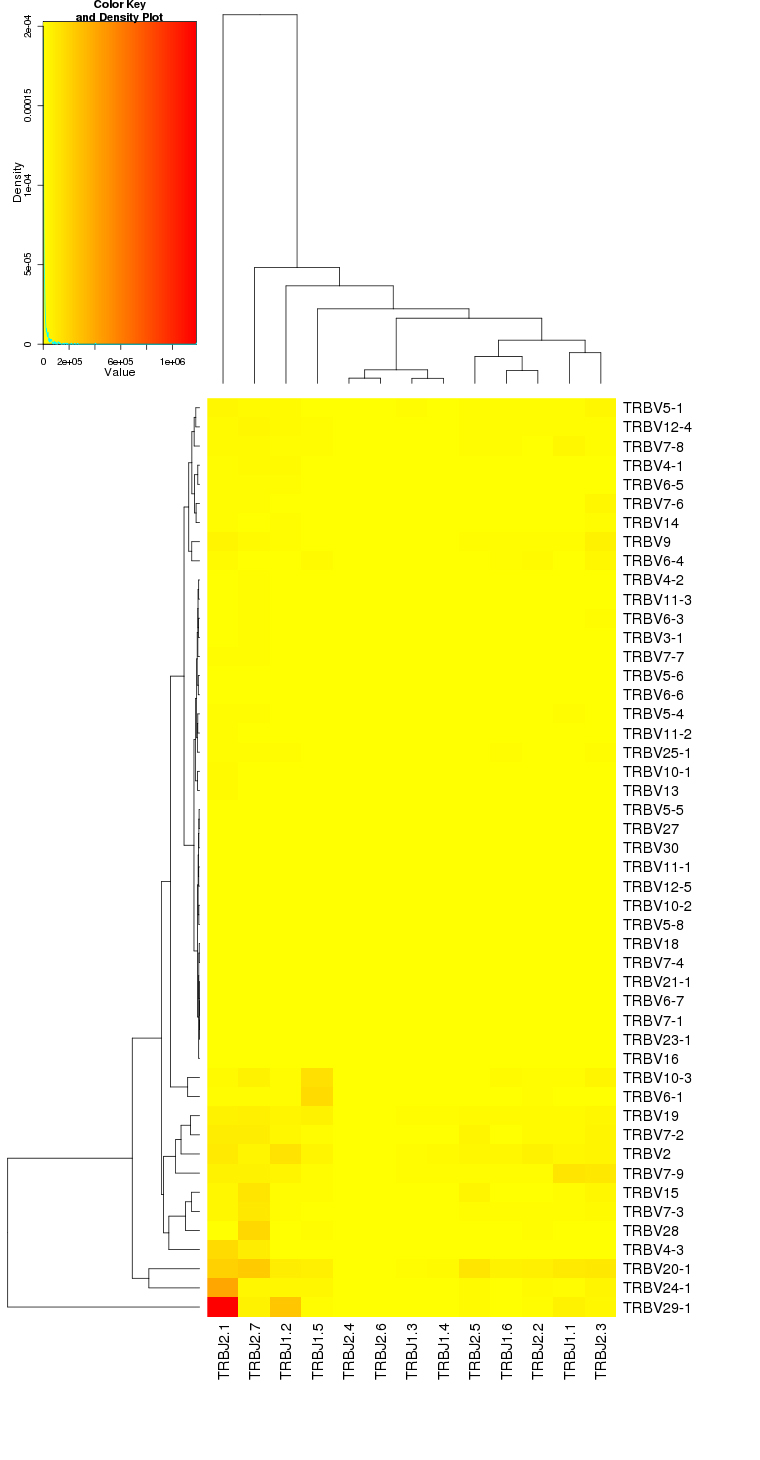

Supplement: Supplemental Digital Content [file medi-95-e02839-s023.jpg]

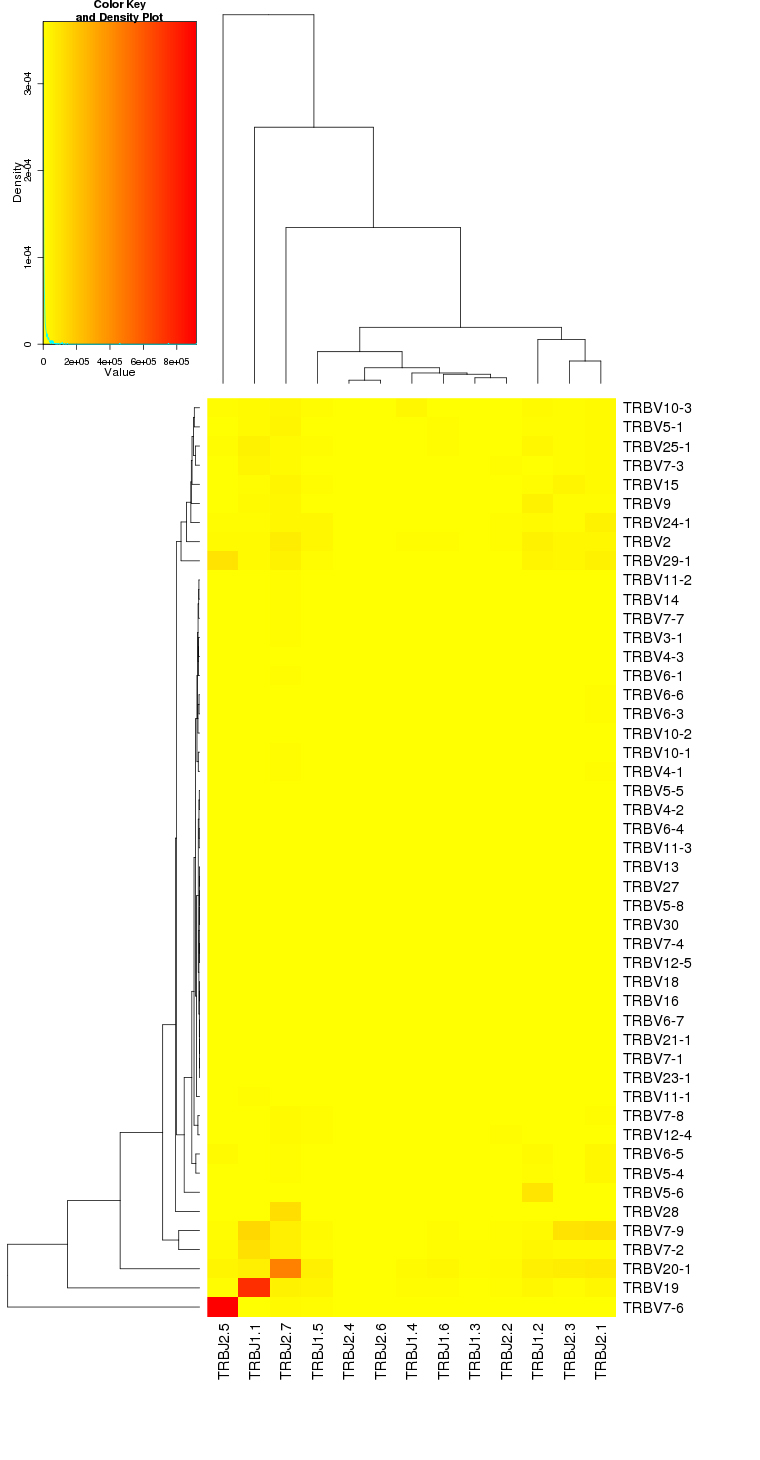

Supplement: Supplemental Digital Content [file medi-95-e02839-s024.jpg]

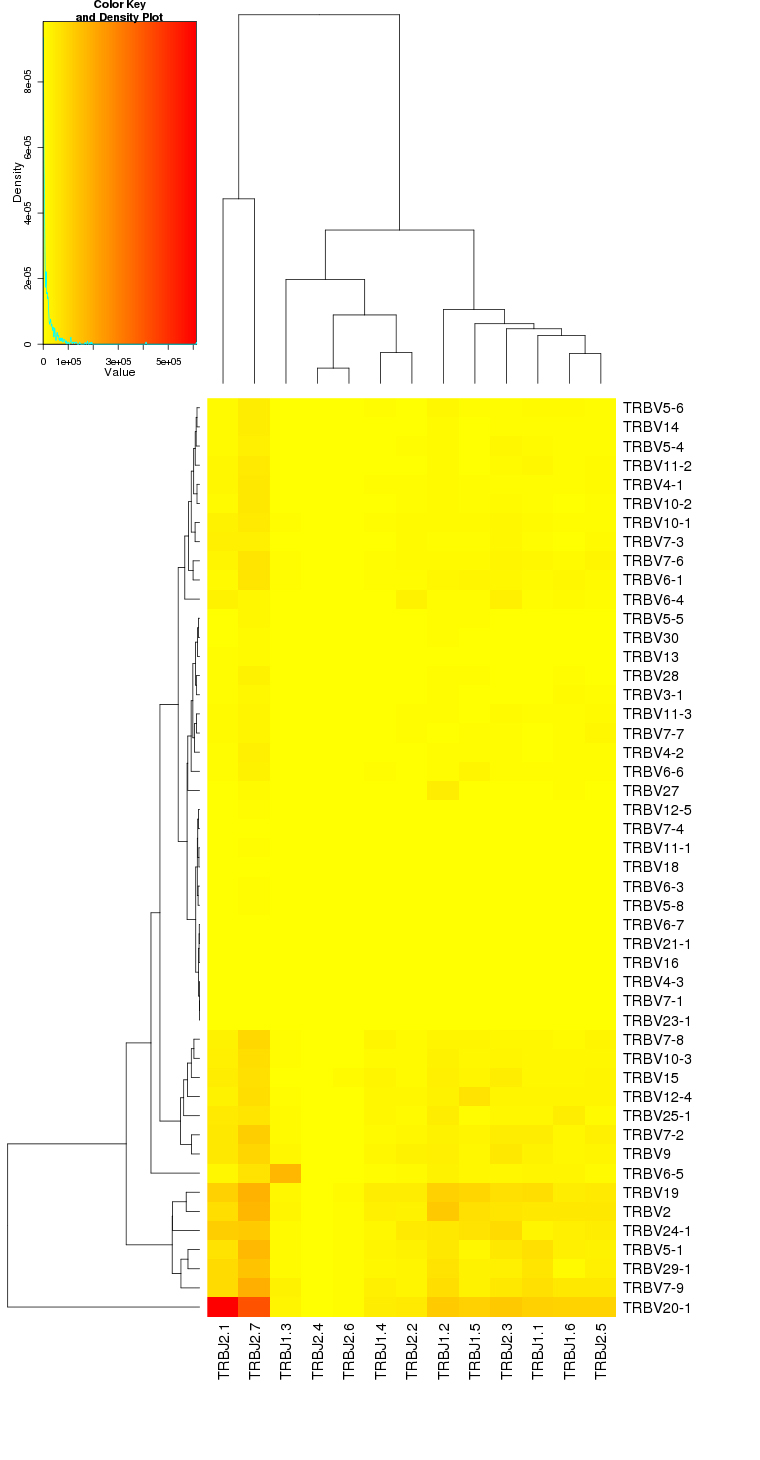

Supplement: Supplemental Digital Content [file medi-95-e02839-s025.jpg]

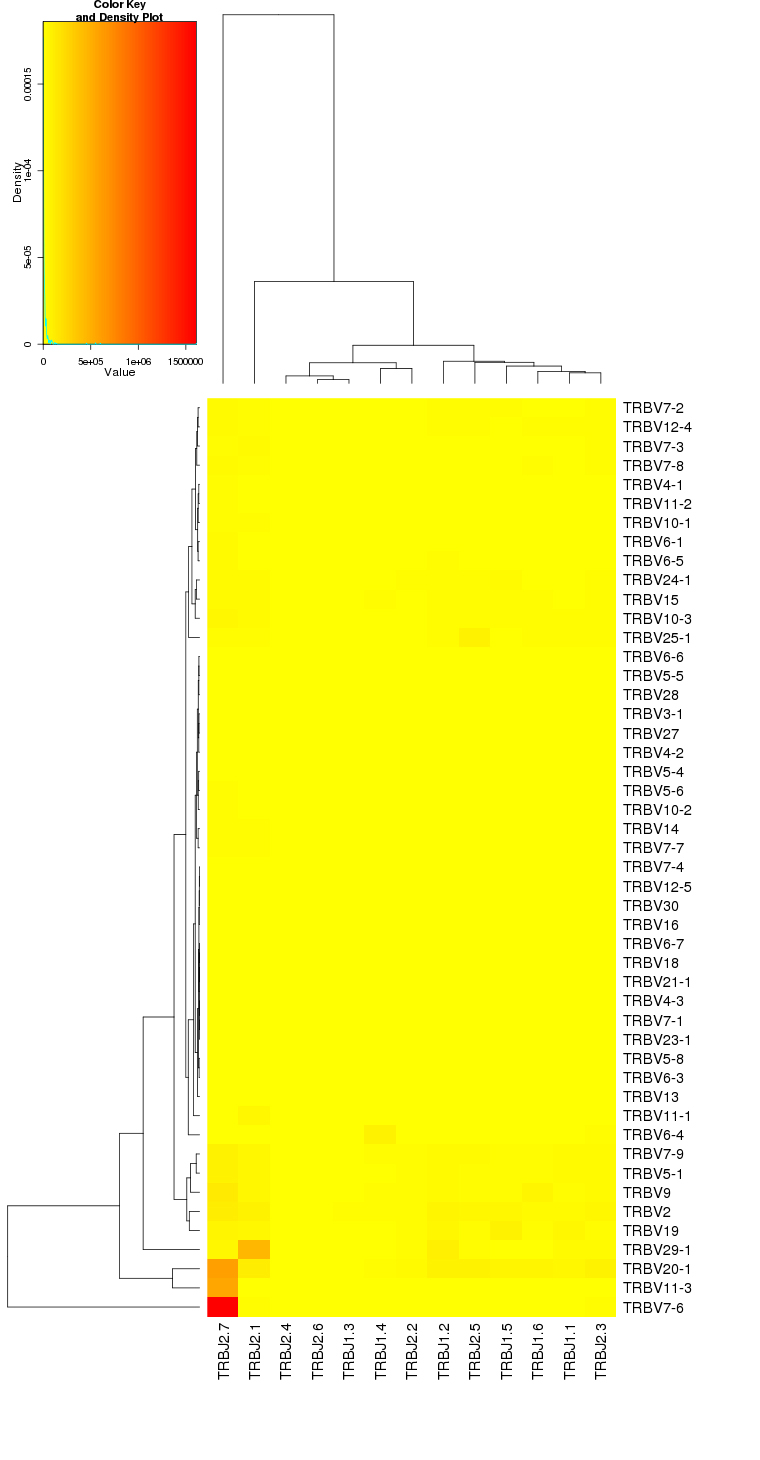

Supplement: Supplemental Digital Content [file medi-95-e02839-s026.jpg]

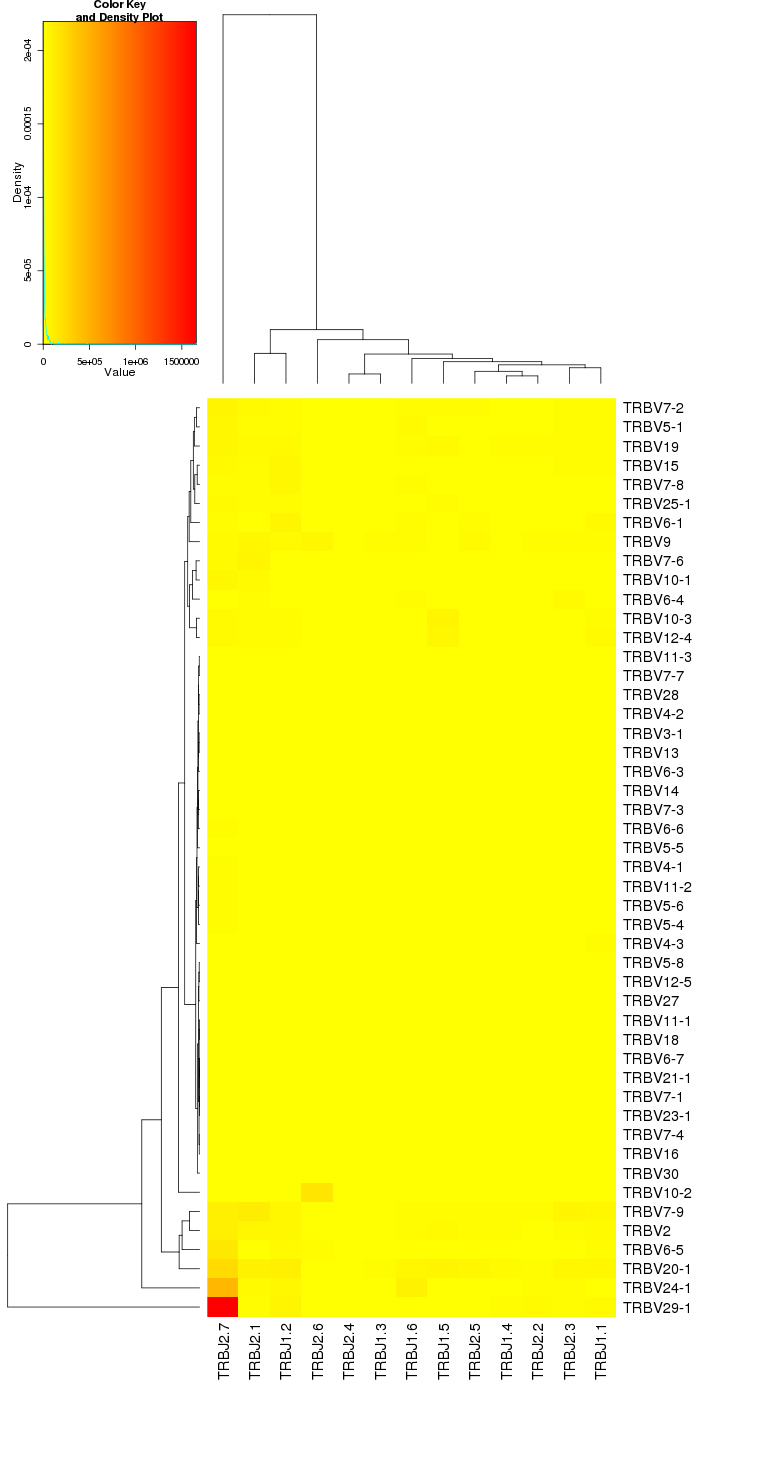

Supplement: Supplemental Digital Content [file medi-95-e02839-s027.jpg]

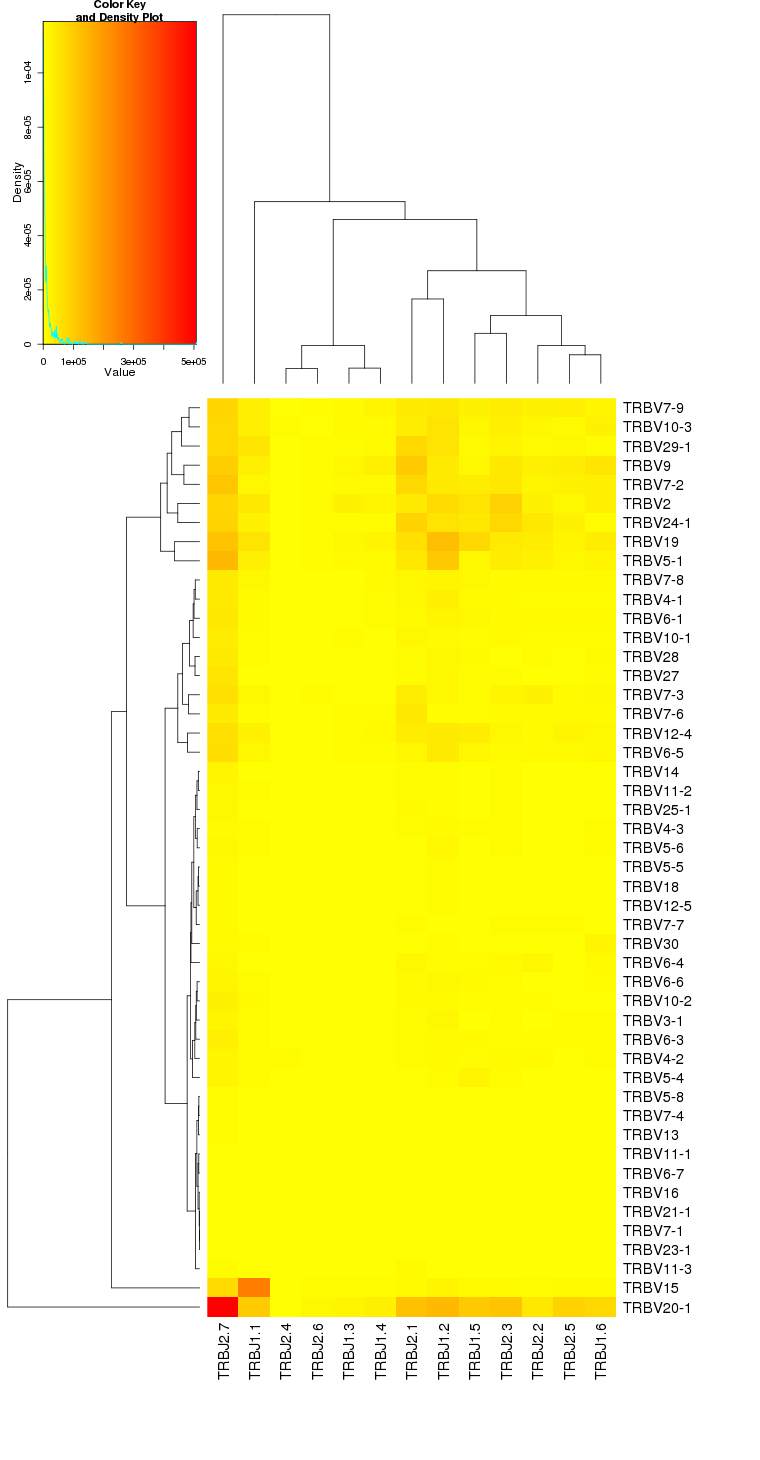

Supplement: Supplemental Digital Content [file medi-95-e02839-s028.jpg]

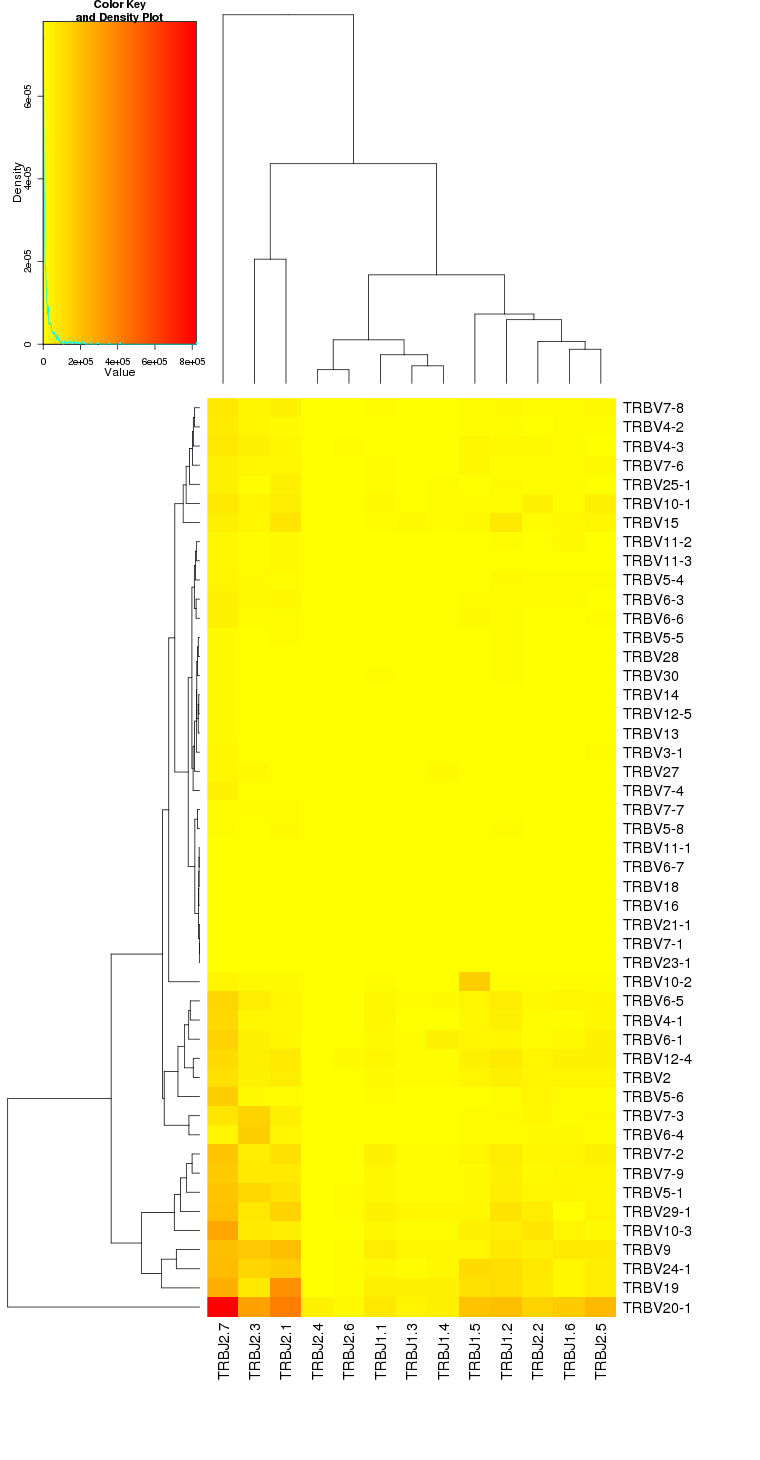

Supplement: Supplemental Digital Content [file medi-95-e02839-s029.jpg]

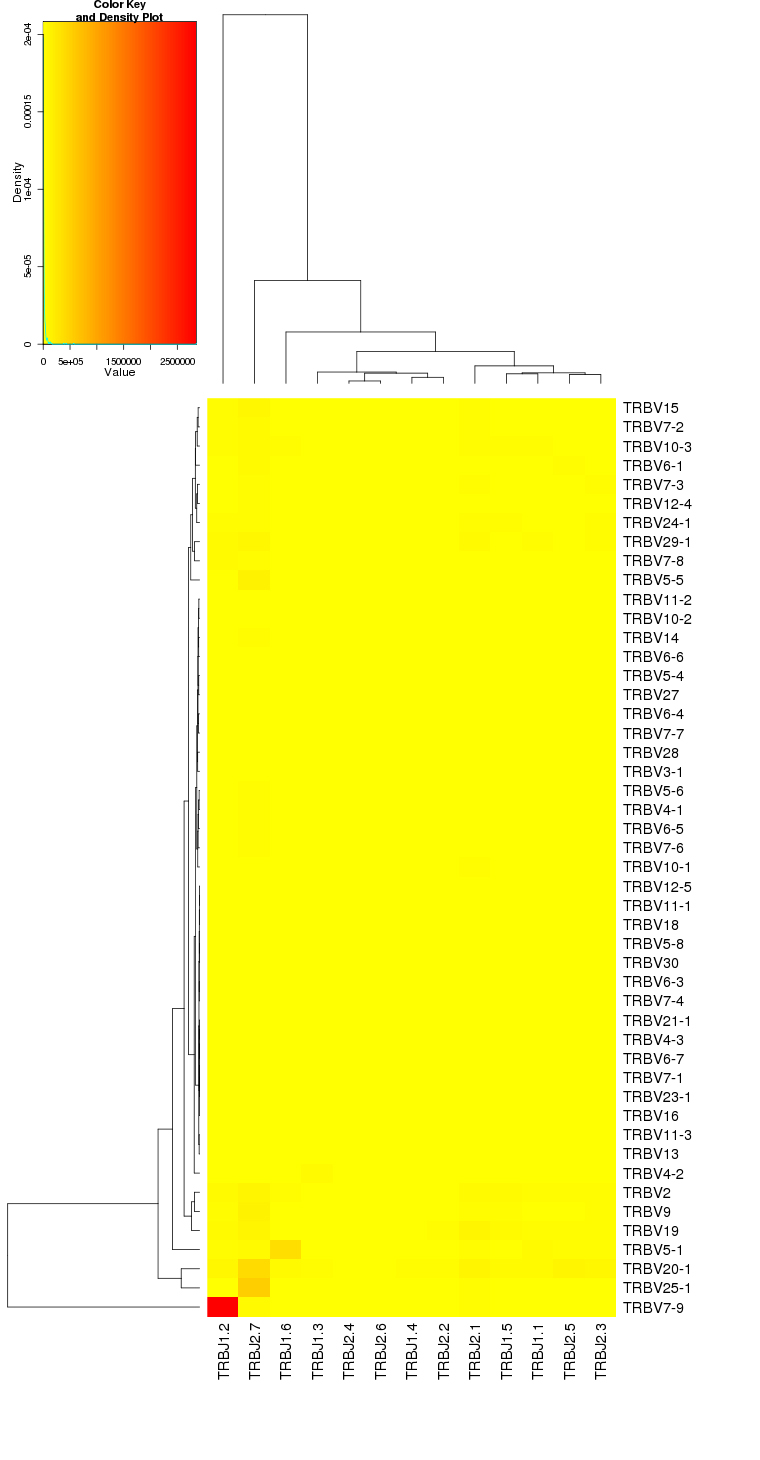

Supplement: Supplemental Digital Content [file medi-95-e02839-s030.jpg]

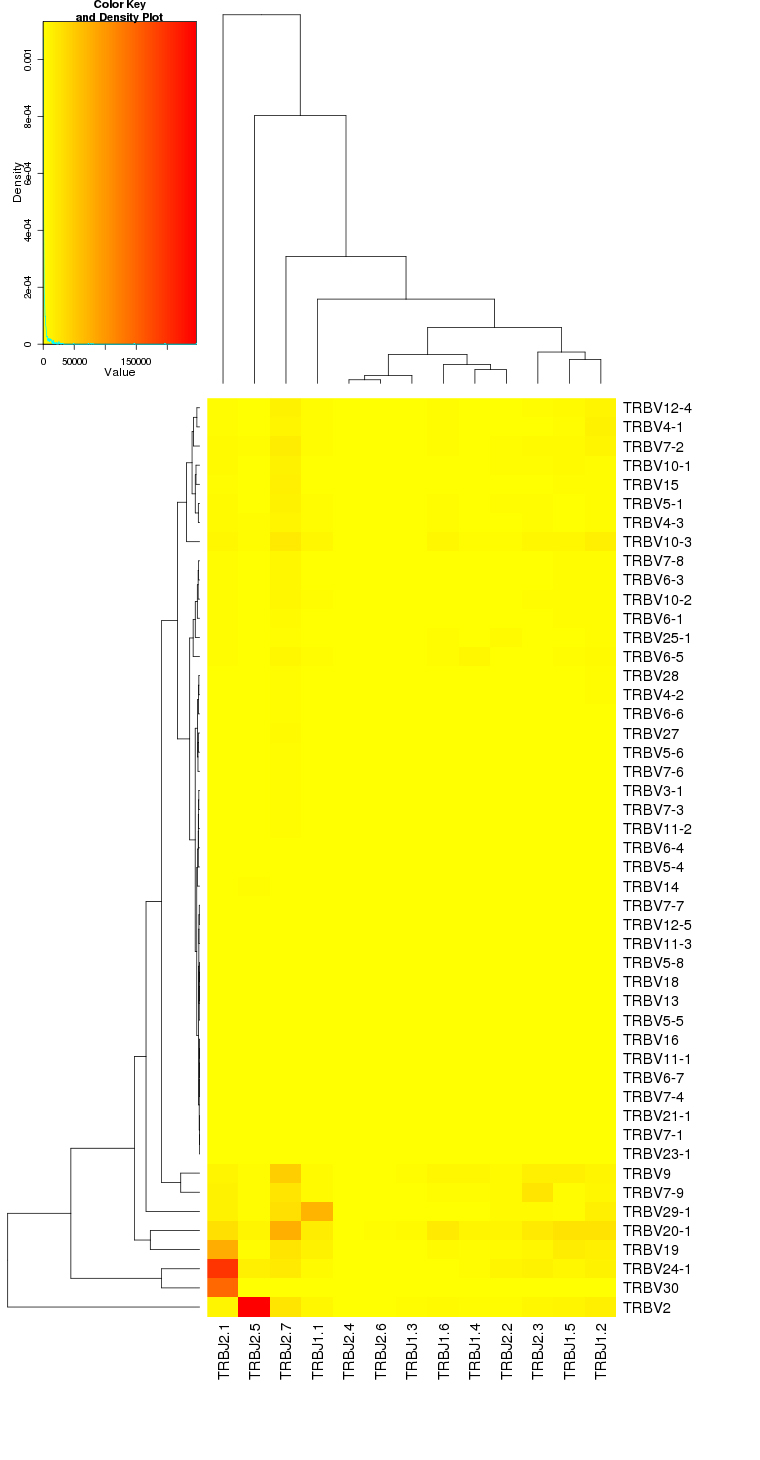

Supplement: Supplemental Digital Content [file medi-95-e02839-s031.jpg]

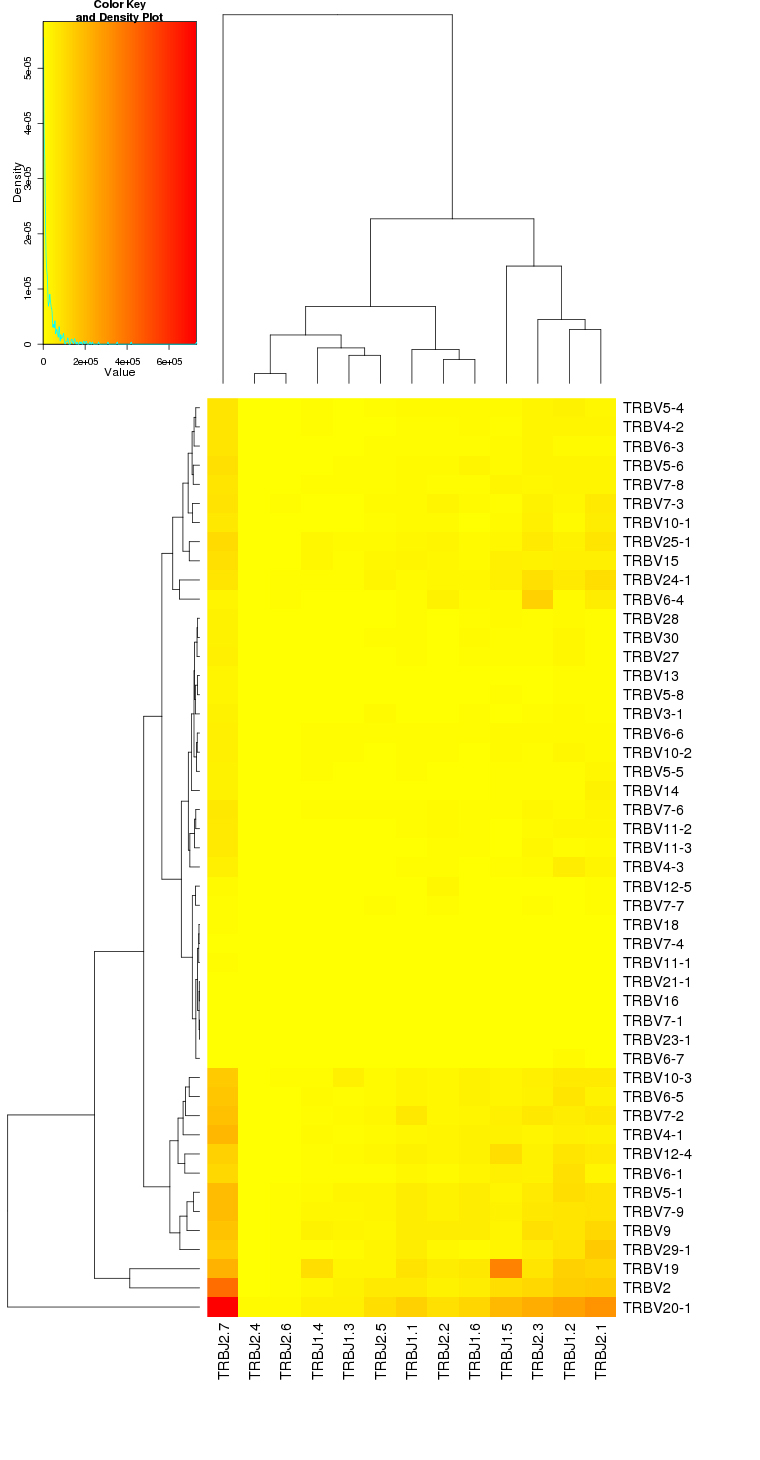

Supplement: Supplemental Digital Content [file medi-95-e02839-s032.jpg]
